# The impact of HIV and antiretroviral therapy on TB risk in children: a systematic review and meta-analysis

**DOI:** 10.1136/thoraxjnl-2016-209421

**Published:** 2017-01-23

**Authors:** P J Dodd, A J Prendergast, C Beecroft, B Kampmann, J A Seddon

**Affiliations:** 1 School of Health and Related Research, University of Sheffield, Sheffield, UK; 2 Blizard Institute, Queen Mary University of London, London, UK; 3 Zvitambo Institute for Maternal and Child Health Research, Harare, Zimbabwe; 4 Centre of International Child Health, Department of Paediatrics, Imperial College London, London, UK; 5 Vaccines & Immunity Theme, MRC Unit The Gambia, The Gambia

**Keywords:** Tuberculosis

## Abstract

**Background:**

Children (<15 years) are vulnerable to TB disease following infection, but no systematic review or meta-analysis has quantified the effects of HIV-related immunosuppression or antiretroviral therapy (ART) on their TB incidence.

**Objectives:**

Determine the impact of HIV infection and ART on risk of incident TB disease in children.

**Methods:**

We searched MEDLINE and Embase for studies measuring HIV prevalence in paediatric TB cases (‘TB cohorts’) and paediatric HIV cohorts reporting TB incidence (‘HIV cohorts’). Study quality was assessed using the Newcastle-Ottawa tool. TB cohorts with controls were meta-analysed to determine the incidence rate ratio (IRR) for TB given HIV. HIV cohort data were meta-analysed to estimate the trend in log-IRR versus CD4%, relative incidence by immunological stage and ART-associated protection from TB.

**Results:**

42 TB cohorts and 22 HIV cohorts were included. In the eight TB cohorts with controls, the IRR for TB was 7.9 (95% CI 4.5 to 13.7). HIV-infected children exhibited a reduction in IRR of 0.94 (95% credible interval: 0.83–1.07) per percentage point increase in CD4%. TB incidence was 5.0 (95% CI 4.0 to 6.0) times higher in children with severe compared with non-significant immunosuppression. TB incidence was lower in HIV-infected children on ART (HR: 0.30; 95% CI 0.21 to 0.39). Following initiation of ART, TB incidence declined rapidly over 12 months towards a HR of 0.10 (95% CI 0.04 to 0.25).

**Conclusions:**

HIV is a potent risk factor for paediatric TB, and ART is strongly protective. In HIV-infected children, early diagnosis and ART initiation reduces TB risk.

**Trial registration number:**

CRD42014014276.

**Key messages**

**What is the key question?**

- What effects do HIV infection and antiretroviral therapy (ART) have on TB risk in children?

**What is the bottom line?**

- HIV infection increases the incidence of TB in children by a factor of around 8, increasing with degree of immunosuppression; ART reduces TB risk by around 70%, with protection continuing to increase over 1–2 years.

**Why read on?**

- TB incidence in HIV-infected children is very high, but no systematic review has quantified the effects of HIV or ART on TB risk in children.

## Introduction

Children are at high risk of progression to TB disease following infection with *Mycobacterium tuberculosis*, particularly children in the first 2 years of life, who often develop non-pulmonary forms of TB.^1^ The variety of presentation, difficulty in obtaining samples for laboratory testing and paucibacillary nature of disease mean that confirming a TB diagnosis in children can be challenging. This adds to difficulties in understanding the natural history and epidemiology of disease. Recent indirect approaches to burden estimation have used mathematical modelling of exposure and disease progression risks to predict paediatric TB incidence.^2^ WHO estimated that in 2014, 1 million children developed TB globally.^3^

Although programmes to prevent mother-to-child transmission (PMTCT) of HIV have reduced new cases of vertically infected infants, coverage is incomplete; in 2013, an estimated 199 000 (170 000–230 000) children were newly infected with HIV.^4^ Children experience more rapid HIV disease progression than adults, making them highly susceptible to opportunistic infections.^5^ Antiretroviral therapy (ART) can restore immune function and has enormously reduced morbidity and mortality among HIV-infected children. In 2015, WHO guidelines were revised, recommending all HIV-infected children should initiate ART, irrespective of clinical disease stage or degree of immunosuppression.^6^ However, ART coverage among children lags behind adults, with only one-third of eligible children currently on ART compared with two-thirds of adults.^4^

In adults, HIV is a known potent risk factor for developing TB, with incident rate ratios (IRR) >5 when averaged across all levels of immunodeficiency.^7^ Evidence synthesis suggests an exponential increase in the IRR with decrease in CD4 T-cell counts.^8^ ^9^ The effect of ART in reducing TB risk in adults living with HIV infection is well described.^10^ This quantitative understanding of the effects of HIV and ART on TB progression has been widely used by modellers, for example, in predicting the impact of HIV interventions on TB incidence.^9^ ^11^ ^12^ In contrast to adults, the impact of HIV infection and ART on TB progression in children is poorly quantified; no systematic reviews have been performed to evaluate this relationship. In the context of revised WHO treatment recommendations, our objective was therefore to systematically review the paediatric HIV/TB literature to quantify the effect of HIV and ART on TB risk in children.

## Methods

This systematic review is reported in accordance with the Preferred Reporting Items for Systematic Review and Meta-Analyses statement^13^ (see online supplementary material). A protocol was registered with PROSPERO (identification number: CRD42014014276) in October 2014.

10.1136/thoraxjnl-2016-209421.supp1 supplementary material

### Cohort definitions

For all studies, the exposure/intervention was HIV infection, with or without ART. We sought studies that either: (i) reported HIV prevalence in children with TB (‘TB cohorts’) or (ii) reported TB disease incidence in cohorts of children with HIV (‘HIV cohorts’). For TB cohorts, the outcome was HIV prevalence, and the comparator was HIV prevalence in control groups of children without TB. For HIV cohorts, the outcome was TB incidence, and the comparison was between subgroups of the same cohort with different levels of immune suppression, or ART exposure (all HIV-infected).

### Search strategy, selection criteria, data extraction

To be eligible, a study had to present empirical data on more than five cases of TB in children (aged <15 years). Studies were excluded if they were not generally representative of paediatric TB cases in that population at that time, for example, studies that only addressed one form of TB; that only comprised hospitalised TB cases; studies explicitly focused on migrant populations or autopsy studies. If the same cohort was published more than once, or individuals were described in more than one cohort, studies/children were only included once. Where explicit use of isoniazid preventive therapy (IPT) was stated, children receiving IPT were excluded. TB cohorts were excluded if the coverage of HIV testing was below 70% among TB cases. HIV cohorts were excluded if data were not reported that could be interpreted as an incidence (ie, number of events and person-time).

MEDLINE and Embase were systematically searched without language, publication or date restrictions using terms designed to capture children and TB and (HIV or ART) on 20 December 2014 (see online supplementary material for full search). A combination of MeSH and EMTREE headings were used with free-text terms to enhance the sensitivity of the search. We further searched the Cochrane Controlled Trials Register and AIDSinfo for relevant studies. All references in review articles found by our database search were included. All citations listed in Google Scholar of the five most cited review articles found by our database search were screened. One additional study in press at the time of our systematic review was brought to our attention after presenting draft results.^14^ Two investigators (PJD and JAS) independently assessed titles and abstracts for inclusion. Full texts were independently assessed for inclusion and study type by PJD and JAS with disagreements resolved by discussion. Two investigators extracted the data for TB cohorts in tandem; PJD extracted the data for HIV cohorts with 25% of data checked by JAS.

For both study designs, the following were recorded: first author, publication year, study years, study country, number of TB cases, age range, number of male children. For TB cohorts, if local TB-free controls were reported in the study, the HIV prevalence among control children (numerator and denominator) was extracted. Other data extracted from the TB cohorts included: the coverage of HIV testing, the numerator and denominator of HIV prevalence in TB cases and the UNAIDS paediatric HIV estimate for corresponding country-year (if available; mid-point and uncertainty range). For HIV cohorts, the following were recorded: TB incidence (with uncertainty range), ART coverage at enrolment, estimate of protective effect of ART against TB (if present) and TB incidence in any ART-related or immune-related stratum (with uncertainty range). Co-trimoxazole use was recorded where described. TB incidence by age band was extracted if present. Some studies recorded immune status using the WHO immunological classification^6^ (not-significant, mild, advanced and severe immunosuppression), whereas others used alternative CD4% or CD4 count categories, which differed between studies. Mid-points and CIs (assumed Poisson exact) were used to infer event numbers and person-time when aggregation was necessary. Some studies excluded incident TB within the first few months on ART, stating that they sought to exclude immune reconstitution inflammatory syndrome reactions and only determine true TB incidence.

Quality of individual studies was assessed using an adapted Newcastle-Ottawa quality assessment tool.^15^ The version for case-control studies was used for TB cohorts; the version for cohort studies for HIV cohorts. For HIV cohorts, the quality for each study as input to each meta-analysis was assessed separately (see online supplementary material pp. 18–21) and reported as low/moderate/high quality (depending on whether few/some/most criteria were met) on domains of selection, comparability and either outcome (for cohort studies) or exposure (for case-control studies).

### Statistical analyses

For TB cohorts, we undertook a random-effects meta-analysis of the OR for HIV prevalence given TB among those studies reporting HIV prevalence in controls. This OR can be interpreted as an IRR for developing TB disease if HIV positive^7^ (see online supplementary material p. 7). We produced funnel plots to assess evidence of publication bias.

For TB cohorts where UNAIDS estimates of national HIV prevalence in the age group aged <15 years were available for the same year and country as the study, we undertook a Bayesian meta-analysis using both the UNAIDS data and control data. This analysis modelled the relationship between UNAIDS HIV prevalence and HIV prevalence in study controls from studies where both were available, and used this relationship to predict individual study ORs where controls were not available. For comparison, a Bayesian version of the meta-analysis of studies with controls was conducted (see online supplementary material pp. 8–11).

For HIV cohorts reporting TB incidence by immune stage, we undertook a random-effects meta-analysis of the IRR for each stratum relative to the ‘not-significant’ WHO immune stage. For HIV cohorts reporting incidence by >1 CD4% category (using the mid-point of the CD4% category in which the incidence was reported), or an analysis of the relation between CD4% and IRR for TB, we undertook a Bayesian meta-analysis to estimate the gradient of logarithmic IRR with respect to CD4% (see online supplementary material pp. 13–15). We averaged the IRR implied by this point estimate over CD4% between 0% and 50% to compare with our IRR from the TB cohort analysis. For HIV cohorts reporting an estimate of the protection against TB from ART as a HR, we performed a random-effects meta-analysis. For HIV cohorts reporting TB incidence by >1 category of time-since-ART-initiation, we fitted a non-linear mixed-effects model to estimate the temporal dependence of ART protection, using the mid-point of the time window in which the incidence was reported to model incidence (see online supplementary material pp. 15–16).

Bayesian techniques were employed where greater flexibility was required over standard software implementations to use all the data*.* All statistical analyses were undertaken using R statistical software (R Core Team. R: A language and environment for statistical computing. R Foundation for Statistical Computing, Vienna, Austria. URL http://www.R-project.org); random-effects meta-analysis was performed using the rma command in the metafor package implementing the restricted maximum-likelihood estimator.^16^

## Results

### Search results

**Figure 1:**
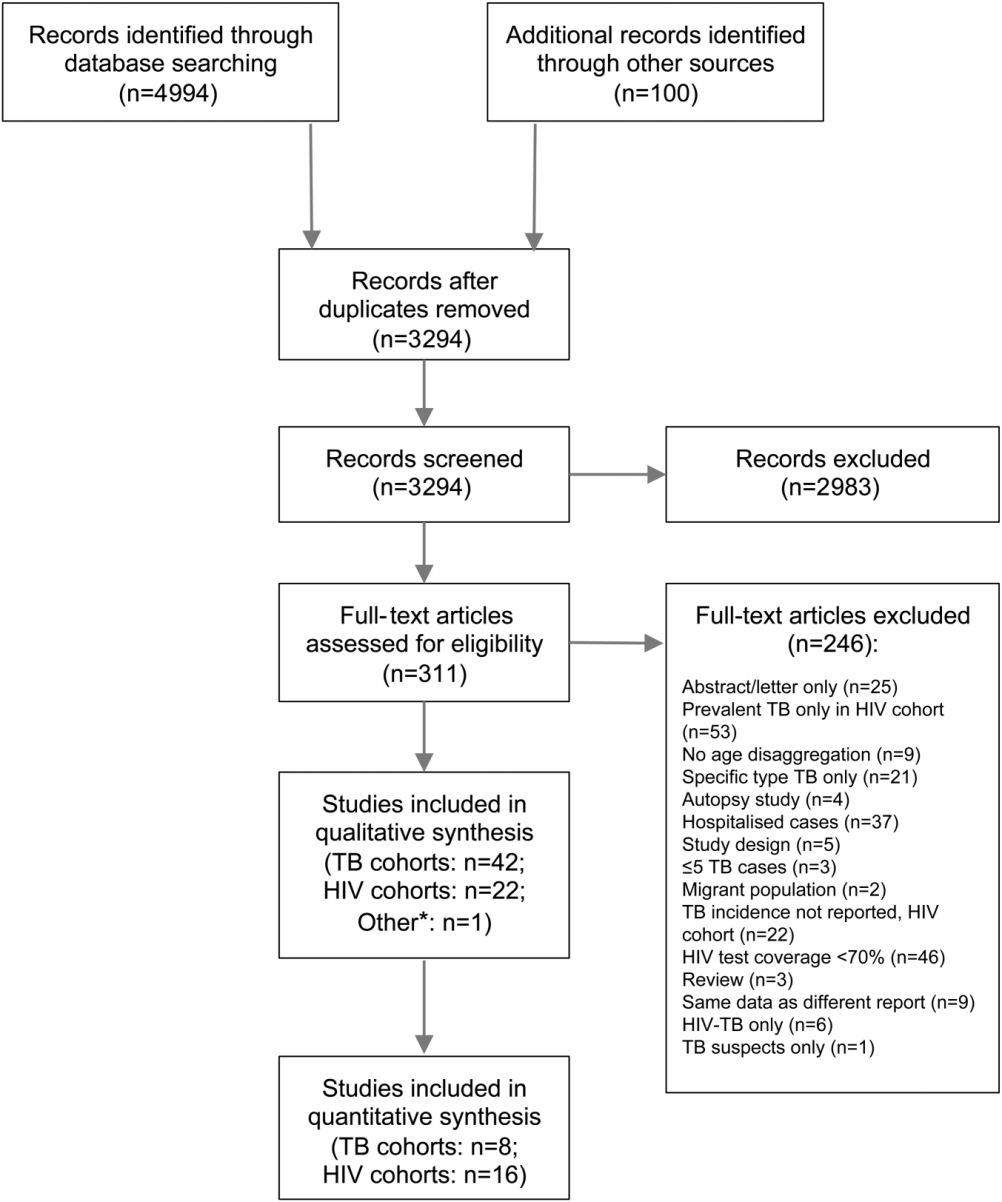
Preferred Reporting Items for Systematic Review and Meta-Analyses flow chart for systematic review (*one study included both a TB and HIV cohort^17^).

The systematic review process is presented in figure 1. Of the 311 full-text articles screened, 65 were included in this review. The most common reasons for exclusion were HIV test coverage <70% for TB cohorts (n=46) and TB incidence not reported for HIV cohorts (n=22). Of the 65 included studies, 42 were classified as TB cohorts (see table 1), 22 were classified as HIV cohorts (see table 2) and 1 study included both a TB and HIV cohort.^17^ Thirty-one of the 42 TB cohorts (74%) and 17 of the 22 HIV cohorts (77%) were from sub-Saharan Africa.

**Table 1:** TB cohorts

First author, year	Country	Years of study	Study description	Control HIV tested	Control with HIV	Children with TB	TB cases tested for HIV	TB cases with HIV	TB cases male	Age range	Quality assessment* (selection/comparability/exposure)
Ade, 2013^18^	Benin	2009–2011	Cross-sectional study reviewing of all children treated for TB in one city over a 3-year period	na	na	182	167	49	88	0–14	C/-/C
Berggren Palme, 2004^19^	Ethiopia	1995–1997	Prospective study recruiting all children investigated for TB at one hospital over a 13-month period	na	na	355	355	54	ns	0–14	B/-/C
Berggren Palme, 2001^20^	Ethiopia	1995–1997	Case-control study, prospectively recruiting all children diagnosed with TB over a 1-year period at one children’s hospital, with a control group recruited concurrently among children undergoing elective surgery	122	2	377	377	47	186	0–14	B/A/A
Bhat, 1993^21^	Zambia	1991–1991	Case-control study, prospectively recruiting all children diagnosed with TB over a 9-month period at one hospital, with a control group recruited from outpatients clinics and among surgical inpatients	134	18	116	96	36	58	0–14	B/C/A
Bobossi-Serengbe, 2005^22^	Central African Republic	1998–2000	Retrospective analysis of all children treated for TB over a 2-year period in one hospital clinic	na	na	284	284	211	153	1–14	C/-/C
Campos-Herrero Navas, 1997^23^	Gran Canaria	1986–1994	Retrospective analysis of all children treated for TB over a 9-year period on the island	na	na	49	49	1	ns	0–14	B/-/C
Cathebras, 1998^24^	Central African Republic	1997–1998	Prospective study in which all patients (adults and children) treated for TB over a 3-month period at one hospital were tested for HIV	na	na	37	37	4	ns	0–14	B/-/C
Chintu, 1993^25^	Zambia	1990–1991	Case-control study of all children treated for TB over an 18-month period in one teaching hospital. Controls were recruited from the emergency department or inpatient surgical wards	242	26	265	237	88	125	0–15	B/C/A
Chintu, 1998^26^	Zambia	1992–1993	Case-control study of all patients (adults and children) presenting with diarrhoea over an 8-month period at one teaching hospital. Cases were HIV-infected with controls HIV-uninfected. Paediatric analysis restricted to children under 5 years	na	na	4	4	3	ns	1–5	C/-/C
Chintu, 1995^27^	Zambia	1990–1991	Prospective study of all children under 5 years admitted to the ward of a teaching hospital. Comparisons made between children with HIV and children without HIV, with respect to proportion with TB	160	15	61	61	42	ns	0–5	B/C/A
Daniel, 2007^28^	Nigeria	1999–2003	Retrospective study of all children diagnosed with TB at one TB centre over a 5-year period	na	na	76	76	8	46	1–14	A/-/A
Edwards, 2007^29^	Democratic Republic of Congo	2002–2003	Retrospective study of all children treated for TB at one hospital over a 1 year period	na	na	110	91	42	ns	0–15	B/-/C
Espinal, 1996^30^	Dominican Republic	1991–1994	Prospective study of all children aged 18–59 months treated for TB at two institutions over a 3-year period	na	na	204	189	11	92	1–5	B/-/C
Feldacker, 2012^31^	Malawi	2008–2010	Retrospective study of all patients (adults and children) treated for TB at one TB treatment centre over a 3-year period	na	na	364	338	148	168	0–14	C/-/C
Gava, 2013^32^	Brazil	1997–2006	Retrospective review of routinely collected data for all children treated for TB in one state over a 10-year period	na	na	356	356	3	183	0–14	C/-/C
Geoghagen, 2004^33^	Jamaica	1999–2002	Retrospective review of all children 0–12 years treated for TB at one hospital over a 4-year period	na	na	26	24	11	16	0–12	C/-/C
Henegar, 2013^34^	Democratic Republic of Congo	2006–2007	Prospective study of all patients (adults and children) starting treatment for TB at 14 clinics over a 17-month period	na	na	830	701	59	398	0–14	C/-/C
Hesseling, 2009^35^	South Africa	2004–2006	Prospective laboratory data collected from 3 hospitals over a 3-year period, used to estimate the incidence of TB in infants (<12 months) with HIV and without HIV	na	na	245	175	53	133	0–1	C/-/C
Hussain, 2007^36^	India	2003–2004	Prospective study of all children admitted to one hospital for the treatment of TB, over a 2-year period	na	na	270	270	23	154	0–15	C/-/C
Iriso, 2005^37^	Uganda	2003	Cross-sectional study of all children aged 2–60 months, investigated for TB at one hospital over a 12-week period	na	na	126	126	62	63	0–5	B/-/C
Jain, 2013^38^	India	2010–2012	Prospective study of all children <5 years investigated for suspected TB, at one hospital over a 20-month period	na	na	26	21	6	15	0–5	C/-/C
Jensen, 2012^39^	Spain	1997–2008	Case-control study. Details of all HIV-infected children (<17 years) hospitalised anywhere in the country over a 12-year period were extracted from a central database. 4 HIV-uninfected controls, matched on age and gender, were extracted for each case. Rates of mycobacterial diseases were compared between cases and controls	na	na	30	30	20	ns	0–17	C/-/C
Llerena, 2010^40^	Colombia	2001–2009	Cross-sectional study of data from one laboratory of all children with culture-confirmed TB diagnosed over an 8-year period	na	na	128	128	7	62	0–14	C/-/C
Luo, 1994^41^	Zambia	1991–1992	Prospective study of all children treated for TB at one hospital over an 8-month period. Controls were children with traumatic injuries, selected from the emergency department or from the surgical wards	167	16	120	110	67	70	0–14	B/C/A
Madhi, 1999^42^	South Africa	1996–1997	Prospective study of all children (2 months to 12 years) treated for TB at hospitals attached to an academic department of paediatrics over a 5-month period	na	na	130	130	52	85	0–12	C/-/C
Mehta, 2011^43^	Tanzania	2005–2007	Randomised controlled trial of multivitamin supplementation in children with TB. All children (6 weeks to 5 years) treated for TB at one clinic were included and randomised over a 30-month period	na	na	255	255	87	139	0–5	B/-/C
Miranda, 2011^44^	Brazil	2000–2006	All cases of paediatric TB recorded on a state register over a 6-year period were included. Matching with the state AIDS database then performed	na	na	411	411	27	191	0–14	C/-/C
Mukadi, 1997^45^	Côte d’Ivoire	1994–1995	Prospective study of all children (0–9 years) diagnosed with TB in two TB centres and two hospitals over a 21-month period	161	0	161	160	31	84	0–9	A/A/A
Berggren Palme, 2002^46^	Ethiopia	1995–1997	Prospective study of all children diagnosed with TB at the outpatient clinic or from the inpatient wards at one paediatric hospital over a 13-month period	na	na	517	517	58	259	0–14	B/-/C
Panigatti, 2014^47^	India	2009–2010	Prospective study of all children (0–12 years) diagnosed with TB at one hospital over an 18-month period, treated using a DOTS regimen	na	na	93	93	7	45	0–12	C/-/C
Rachow, 2012^48^	Tanzania	2008–2010	Prospective study of all children diagnosed with TB at one research facility over a 31-month period	na	na	164	164	84	86	0–14	C/-/C
Ramos, 2010^49^	Ethiopia	2007	Retrospective data collection from TB registers and treatment cards of all patients (adults and children) treated for TB over a 10-year period at one private hospital†	na	na	187	158	149	ns	0–14	C/-/C
Rose, 2012^50^	Tanzania	2008–2010	Prospective study of all children with suspected TB at one district hospital over a 27-month period	93	22	33	33	18	24	0–14	A/C/A
Sassan-Morokro, 1994^51^	Côte d’Ivoire	1989–1990	Prospective study of all children diagnosed with TB at two outpatient treatment centres over an 18-month period	na	na	289	289	34	ns	1–14	B/-/C
Schaaf, 2014^52^	South Africa	2009–2011	Retrospective study of all children (<13 years) with culture-confirmed TB at one teaching hospital over a 2-year period	na	na	340	288	63	177	0–13	C/-/C
Seddon, 2012^53^	South Africa	2007–2009	Retrospective study of all children (<13 years) with culture-confirmed TB at one teaching hospital over a 2-year period	na	na	294	217	63	156	0–13	C/-/C
Shahab, 2004^54^	India	1999–2000	Prospective study of all children (<12 years) treated for TB in the outpatient and inpatient departments of one tertiary hospital over a 17-month period	na	na	250	250	5	174	0–12	C/-/C
Thomas, 2014^55^	South Africa	2009–2010	Prospective study of all children (6 months to 12 years) with possible probable or confirmed TB recruited from outpatient and inpatient settings of one district hospital over a 17-month period	na	na	33	26	17	19	0–12	B/-/C
Yassin, 2011^56^	Ethiopia	2009	Cross-sectional study of all children (1–15 years) with TB symptoms and a TB source case who were investigated at two health centres and one hospital. TB-exposed but asymptomatic and unexposed children were also recruited as controls.	153	3	164	141	14	105	1–15	A/C/B
Kwara, 2016^57^	Ghana	2012–2014	Prospective pharmacokinetic study of all children (3 months to 14 years) starting treatment for TB disease at one teaching hospital over a 2-year period	na	na	62	62	28	32	0–14	C/-/C
López-Varela, 2015^58^	Mozambique	2011–2012	Prospective study from one heath district where all children (<3 years) with presumptive TB were recruited over a 12-month period	na	na	32	32	18	15	0–3	B/-/C
Perfura Yone, 2012^59^	Cameroon	2005–2010	Retrospective study all children (<15 years) diagnosed with TB and treated as inpatients at one hospital over a 5½-year period	na	na	101	101	25	50	0–15	B/-/C

*Adapted Newcastle-Ottawa score for case-control studies with some questions (and the comparability domain) not applicable to studies without controls: A=high quality; B=moderate quality; C=low quality; see online supplementary material pp. 18–19, 24–25.

†Data from years with high enough HIV testing rates used; age range obtained where stated, for eligibility otherwise.

na, not applicable; ns, not stated.

**Table 2:** HIV cohorts

First author, year	Country	Years of study	Study description	Number of TB cases	Number in cohort	Patient-years	TB incidence, per cent per year (95% CI)	ART at enrolment (%)	Number male	Age range	Quality, TB incidence (selection/outcomes)*	Quality, immunosuppression (selection/comparability/outcomes)*	Quality, CD4% (selection/comparability/outcomes)*	Quality, time-on-ART (selection/comparability/outcomes)*	Quality, ART protection (selection/comparability/outcomes)*
Abuogi, 2013^60^	Kenya	2009–2010	Prospective cohort study of HIV-infected children (6 weeks to 14 years, some on ART and some not) followed-up for the incidence of clinically diagnosed TB. Study at two urban clinics with children recruited over a 2-year period	10	686	720	1.39 (0.75 to 2.58)	74	322	0–14	B/A	B/C/A	na	na	na
Alarcón, 2012^61^	Brazil	2002–2007	Prospective cohort study of HIV-infected children (<21 years) at 17 sites in Latin America; children recruited over a 5-year period and followed for the incidence of a number of opportunistic infections, including TB	7	731	2523	0.28 (0.07 to 0.48)	67	325	0–22	B/B	na	na	na	na
Auld, 2014^62^	Côte d'Ivoire	2004–2008	A nationally representative retrospective cohort study of children (<15 years) starting ART over a 5-year period at 29 facilities	56	1960	4190	1.34 (1.03 to 1.74)	100	1058	0–14	C/C	na	C/A/C	B/C/C	na
Bakeera-Kitaka, 2011^63^	Uganda	2003–2006	Retrospective cohort study of children and adolescents starting ART over a 3½-year period at one specialist clinic	311	1806	1690	18.4 (17.1 to 20.3)	100	932	ns	C/C	na	na	B/C/C	B/A/C
Braitstein, 2009^64^	Kenya	2001–2007	Retrospective observational study of all children (<13 years) enrolled in a network of HIV clinics over a 5-year period	765	6301	4368	17.6 (16.3 to 18.8)	19	3144	0–13	C/C	na	na	B/C/C	B/A/C
Brennan, 2014^65^	South Africa	2004–2011	Retrospective cohort of all children (<19 years) on ART enrolled over a 7-year period at 12 urban clinics	113	3329	2828	4.0 (3.3 to 4.8)	100	1608	0–18	C/C	na	C/A/C	B/A/C	na
Ciaranello, 2014^66^	East Africa	2002–2008	All HIV-infected infants (<12 months) enrolled over a 6-year period at 7 sites across 3 countries	128	847	738	17.4 (14.5 to 20.6)	0	418	<1	C/C	na	C/C/C	na	na
Crook, 2016^14^	Uganda, Zimbabwe	2007–2012	Randomised controlled trial assessing different monitoring strategies for providing ART. All HIV-infected children (3 months to 17 years) enrolled from 4 centres in 2 countries over an 18-month period	69	969	3632	1.9 (1.5 to 2.4)	100	488	0–17	B/B	na	B/A/B	A/C/B	na
Curtis, 2012^67^	Eight countries	2002–2010	Data from 25 HIV treatment centres in 8 countries collected prospectively over an 8-year period. Adults and children included if starting ART at one of the centres. Data from children (<15 years) disaggregated from adults	140	3946	1687	8.3 (8.1 to 8.6)	100	ns	0–15	C/C	na	na	B/C/C	na
Dankne, 2001^68^	USA	1988–1998	Data from 13 studies over a period of 10 years with children (<21 years) included. From pre-ART period. Wide range of opportunistic infections assessed	27	3331	6750	0.4 (0.3 to 0.6)	0	1852	0–21	C/C	na	C/C/C	na	na
De Beaudrap, 2013^69^	Burkina Faso/Côte d'Ivoire	2006–2007/2000–2004	Pooled data from a clinical trial (recruited over 1 year) and observational cohort (recruited over 4 years). Children (15 months to 15 years) enrolled at initiation of ART and followed for 2 years	10	188	355	2.9 (1.4 to 5.1)	100	66	ns	C/A	na	C/C/A	B/C/A	na
Edmonds, 2009^70^	Democratic Republic of Congo	2004–2008	Prospective study of children (<18 years) enrolled from one hospital clinic over a 3½-year period. All children ART-naïve at baseline with some starting ART during follow-up	81	364	596	13.6 (10.8 to 16.9)	0	172	0–18	B/B	B/C/B	na	A/C/B	A/A/B
Gray, 2014^71^	South Africa	2005–2011	Control (placebo) arm of a randomised controlled trial of isoniazid preventive therapy. Children (over 8 weeks) recruited over a 4-year period from three sites	7	82	240	2.9 (1.2 to 6.0)	100	42	ns	A/B	na	na	na	na
Kouakoussui, 2004^72^	Côte d'Ivoire	2000–2003	All children recruited from one clinic and followed-up for incident TB. All children in the study initiated ART at some point in the study to provide pre-ART and post-ART TB incidences	7	282	206	3.4 (1.5 to 6.7)	65	153	1–16	C/B	na	C/C/C	na	na
Li, 2013^73^	Tanzania	2004–2011	Prospective study of all children (<15 years) from 28 clinics recruited over a 7-year period. ART started based on local guidelines over follow-up	376	5040	7221	5.2 (4.7 to 5.8)	51	2460	0–14	B/C	B/A/C	na	A/A/C	A/A/C
Martinson, 2009^74^	South Africa	1994–2006	Retrospective cohort of children (<15 years) recruited from four clinics over a 12-year period. Incidence determined for the period pre-ART and post-ART	281	1132	1713	12.3 (9.8 to 12.6)	2	563	0–14	B/C	na	na	na	A/A/C
Prasitsuebsai, 2014^75^	SE Asia	1993–2009	Retrospective and prospective cohort of HIV-infected children (≤18 years) from 14 clinics in 5 countries in SE Asia. Recruited over a 16-year period. Wide range of OIs assessed. Period covered before ART, during monotherapy or dual-therapy and on HAART	477	2280	1826	21.5 (19.4 to 23.7)	7	1136	0–18	C/C	na	na	na	na
Thomas, 2000^76^	USA (New York City)	1989–1995	Retrospective study in HIV-exposed and HIV-infected children enrolled in a longitudinal study. TB cases determined by review of clinical records and by linkage to local TB registry. Registry data assessed for a 6-year period	45	1426	7384	0.61 (0.44 to 0.82)	0	475	0–12	B/B	na	na	na	na
Walters, 2008^77^	South Africa	2003–2005	Chart review of all children (<13 years) starting HAART at one hospital over a 3-year period and followed-up until the end of the study period for incident TB. Cases defined as pre-HAART (if in the 9 months prior to HAART initiation) or post-HAART	10	290	546	25.1 (21.1 to 29.6)	0	142	0–14	C/A	na	na	na	na
Walters, 2014^78^	South Africa	2003–2010	Retrospective cohort of all children (<2 years) starting HAART at one hospital over an 8-year period. Children divided into those developing TB-IRIS (developing TB with 3 months of starting HAART) and those with incident TB (developing TB after 3 months)	55	341	855	25.3 (22.0 to 28.9)	0	161	<2	B/C	na	na	A/C/C	na
Yirdaw, 2014^79^	Ethiopia	2007–2010	Retrospective cohort study of the electronic records of all patients (adults and children) starting ART over a 3-year period in five hospitals in one region of Ethiopia. Patients followed until the end of the study period and the interventions of ART and IPT evaluated. Incidence stratified by age enabling extraction children (<15 years)	23	475	1099	2.1 (1.3 to 3.1)	ns	ns	0–15	B/C	na	na	na	A/C/C
Zar, 2007^80^	South Africa	2003–2004	Double-blind randomised controlled trial evaluating the impact of IPT on TB incidence in HIV-infected children (aged older than 8 weeks). Children recruited from two sites with the trial stopped after 17 months. Data on TB incidence taken from placebo arm	13	131	56	23.4 (12.4 to 50.0)	8	74	0–14	C/C	na	na	na	na

*Adapted Newcastle-Ottawa tool for cohort studies: A=high quality; B=moderate quality; C=low quality; see online supplementary material pp. 19–21, 26–27.

ART, antiretroviral therapy; HAART, highly-active antiretroviral therapy; IPT, isoniazid preventive therapy; IRIS, immune reconstitution inflammatory syndrome; OIs, opportunistic infections; na, not applicable; ns, not stated.

Of the TB cohorts, 8 included HIV prevalence in controls without TB and could be used in the random-effects meta-analysis; 35 had relevant UNAIDS estimates and could be included in the Bayesian meta-analysis, including all 8 studies with controls.

Of the HIV cohorts, 7 could be included in the meta-analysis of CD4% influence, 3 could be included in the analysis of the influence of immunological staging, 10 were included in the analysis of time on ART and 6 were included in the pooled estimate of ART efficacy against TB. Only three HIV cohorts reported co-trimoxazole use.^14^ ^67^ ^80^

HIV prevalence in TB cohorts ranged between 5% and 94% (see online supplementary material p. 5). TB incidence in HIV cohorts ranged from 0.3 to 25.3 per 100 person-years (see online supplementary material p. 6). Insufficient data were available in either group of cohorts to stratify results by age.

### Quality assessment

TB cohorts were of low and moderate quality for our analysis (table 1). Studies without controls could not be classed higher than moderate quality on selection and low quality on exposure. Controls were often drawn from clinic populations in the same context. In around half of studies, TB was defined only by a decision to treat.

HIV cohorts were typically of low quality for our analyses (table 2). Most studies were clinic-based and not necessarily community-representative; prevalent TB was not always ruled out at baseline; there were often shortcomings in follow-up or case definitions for TB.

### Meta-analyses for TB cohorts

**Figure 2:**
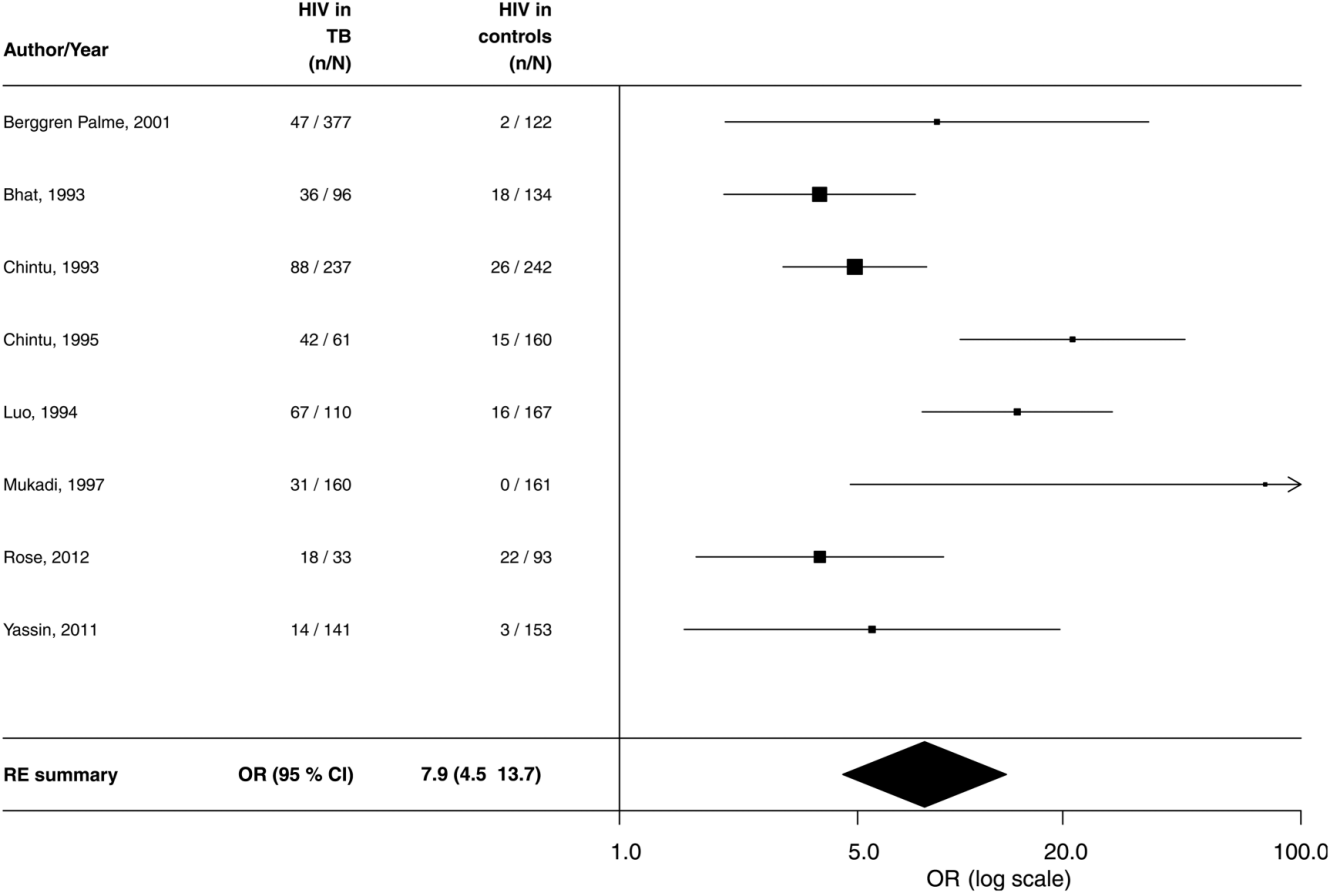
Forest plot for meta-analysis of HIV risk in children aged <15 years with prevalent TB—studies with controls (I^2^=69.8%). RE, random effects.

The random-effects meta-analysis of TB cohorts for the odds of having HIV in TB cases included eight case-control studies (figure 2). In total, 1215 TB cases and 1232 non-TB controls were included. The pooled estimate for the OR was 7.9 (95% CI 4.5 to 13.7), and the I-squared heterogeneity statistic (I^2^) was 69.8% (see figure 2). A funnel plot corresponding to this analysis is presented in online supplementary material p. 8.

**Figure 3:**
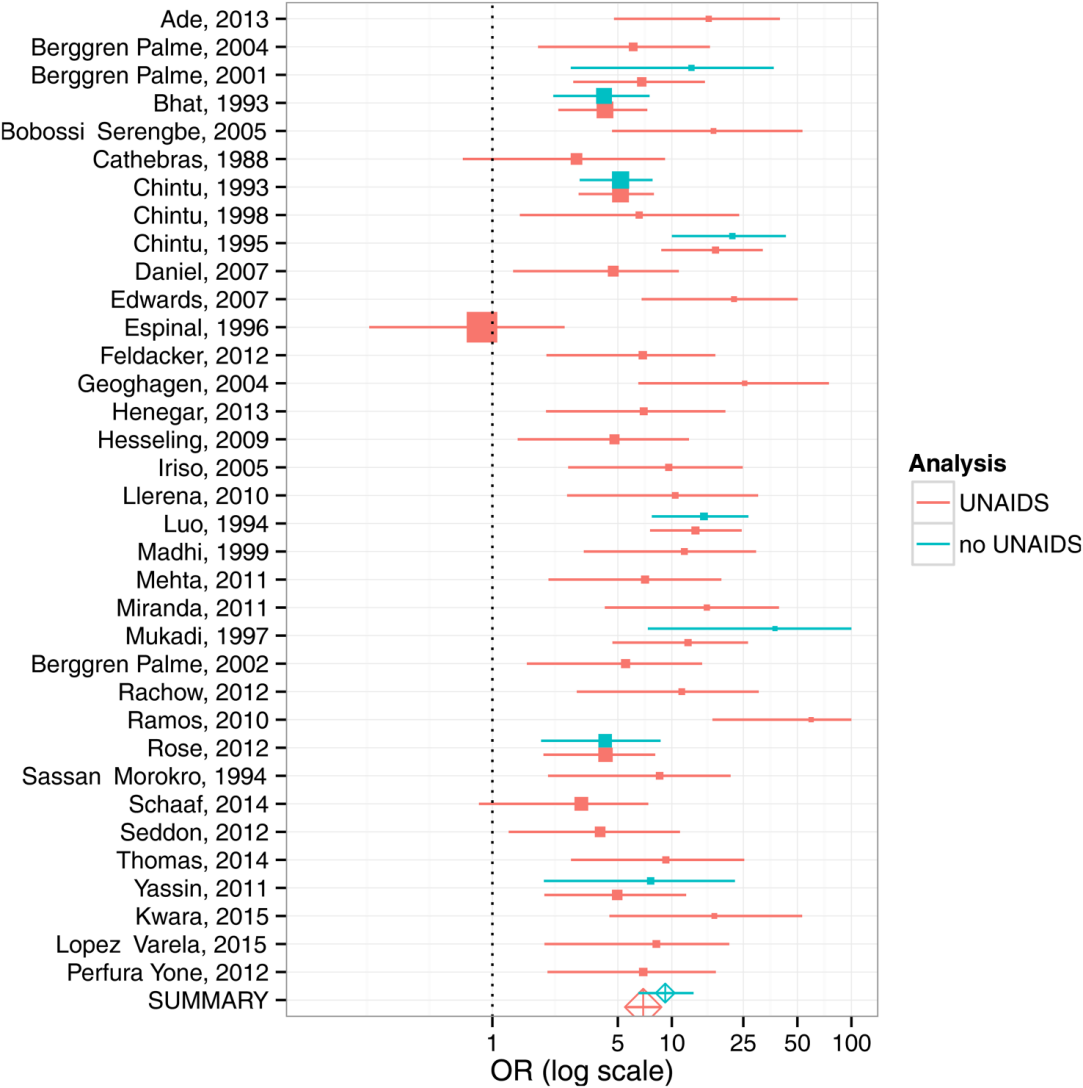
Forest plot for Bayesian meta-analysis of HIV risk in children aged <15 years with prevalent TB. Where studies lacked their own controls, UNAIDS national HIV prevalence data were used to model HIV prevalence in controls based on those studies with both controls and UNAIDS estimates (red). Meta-analyses for studies with controls only are shown in blue; meta-analyses for studies using UNAIDS estimates of paediatric HIV prevalence are shown in red.

The Bayesian meta-analysis included 35 studies (including all 8 with controls) and produced a pooled estimate of the OR for HIV among children with TB of 7.0 (95% credible interval (CrI): 5.7–8.5), see figure 3. This analysis also found the paediatric HIV prevalence in controls to be substantially higher than national UNAIDS estimates of HIV prevalence in the age group aged <15 years, with an OR of 7.3 (95% CrI: 5.9 to 8.8). For studies that lacked explicit control groups (indicated in red in figure 3), this analysis also predicted what the ORs would have been for HIV prevalence in TB cases versus HIV prevalence in a putative control group of comparable children without TB. The pooled estimates of effect for the analyses with and without the UNAIDS data were similar (see online supplementary material p. 11).

### Meta-analyses for HIV cohorts

**Figure 4:**
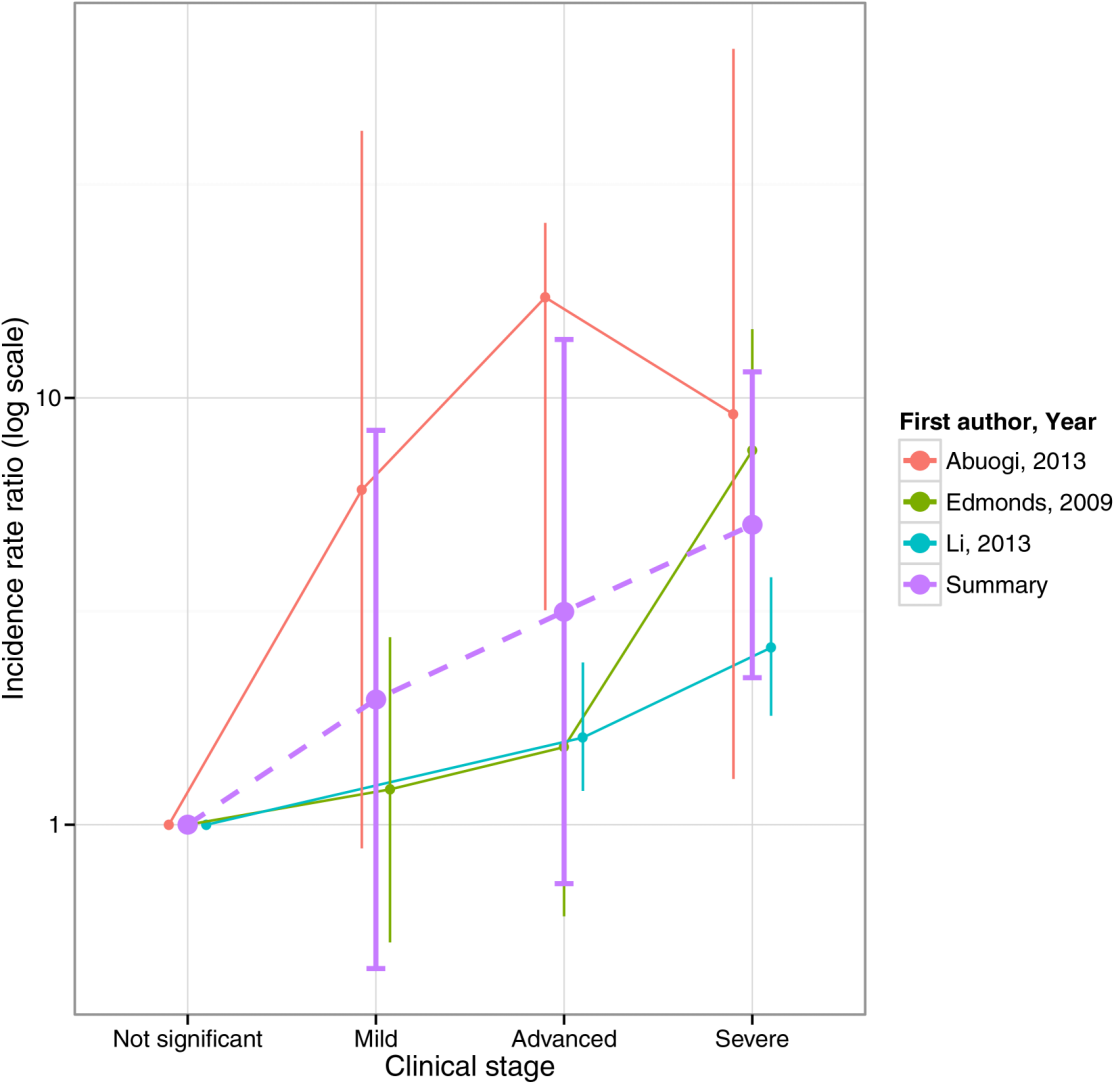
Relative TB incidence in children aged <15 years with HIV by WHO immunological staging (I^2^=87.1%).

**Figure 5:**
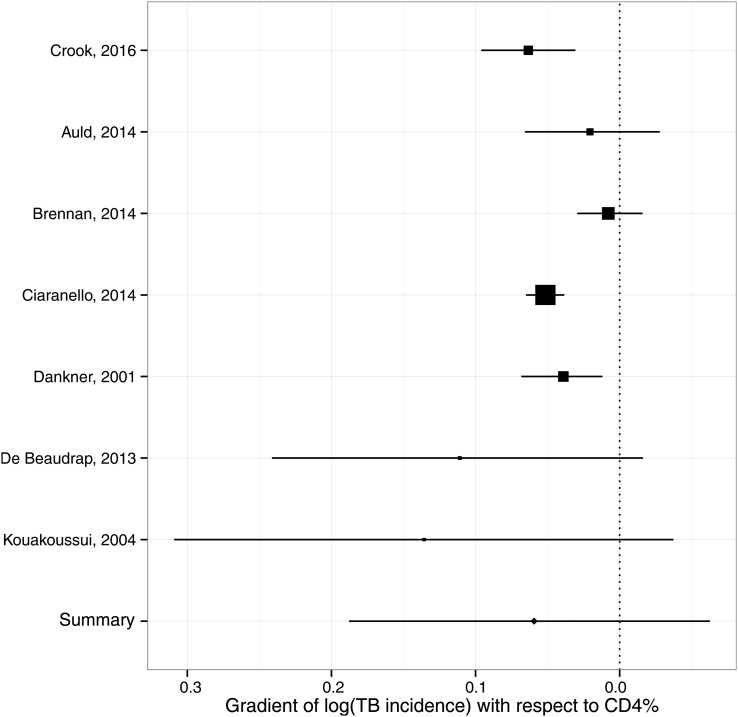
Forest plot for meta-analysis of relation between incidence rate ratio for TB incidence and CD4% in children aged <15 years.

**Figure 6:**
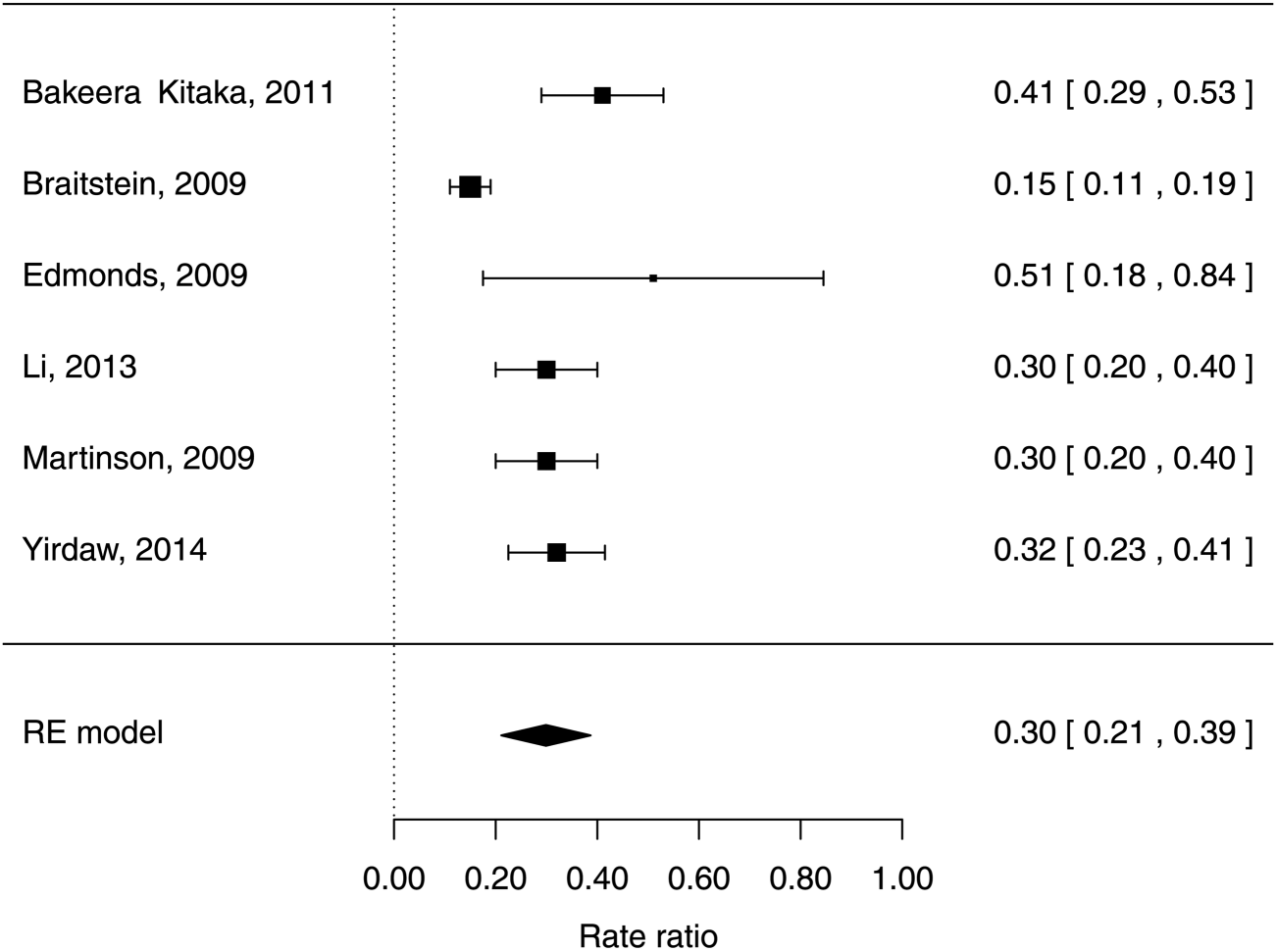
Forest plot of protection on antiretroviral therapy against TB incidence in children <15 years with HIV infection (I^2^=79.0%). RE, random effects.

For HIV cohorts, the random-effects meta-analysis of TB incidence in children with severe compared with non-significant immunosuppression according to the WHO categorisation gave an IRR of 5.0 (95% CI 4.0 to 6.0) with I^2^*=*87.1% (see figure 4). The Bayesian meta-analysis of the gradient of the logarithmic IRR with respect to CD4% yielded a pooled estimate of −0.063 (95% CrI: −0.188 to +0.063), corresponding to a reduction in IRR of 0.94 (95% CrI: 0.83 to 1.07) per one percentage-point increase in CD4% (see figure 5). This point estimate implies a mean IRR of 7.1 over CD4% ranging between 0% and 50%. The random-effects meta-analysis of protection from ART yielded a pooled HR estimate of 0.30 (95% CI 0.21 to 0.39) with I^2^*=*79% (see figure 6).

**Figure 7:**
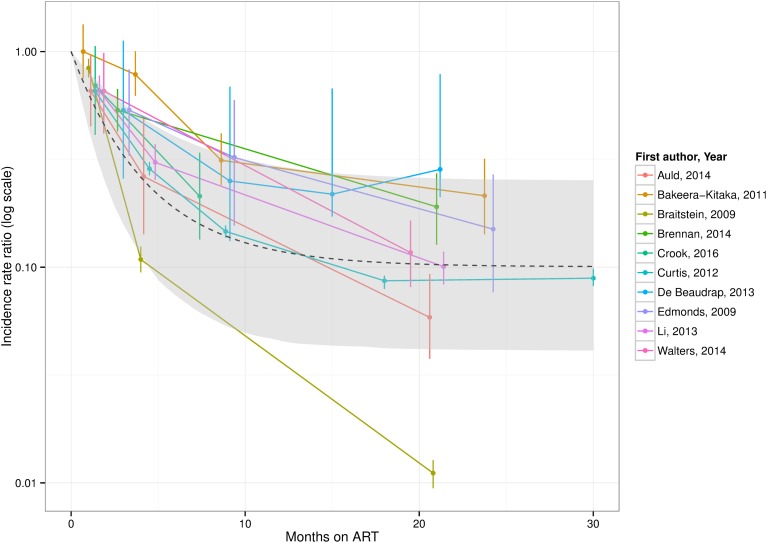
Meta-regression of protection from TB incidence in children <15 years by time-on-antiretroviral therapy (ART), and realigned incidence estimates from studies.

The non-linear mixed-effects regression for the protection from ART by time-since-initiation estimated a rapid initial decline in incidence over the first year, reaching a plateau as protection from ART fully establishes over around 2 years at a pooled asymptotic HR of 0.10 (95% CI 0.04 to 0.25), giving a mean HR over the first 30 months on ART of approximately 0.25 (see figure 7).

## Discussion

In this systematic review, we identified cohorts of children with TB and cohorts of children with HIV. We observed a high but variable prevalence of HIV infection in the TB cohorts, and a very high TB incidence in the HIV cohorts. Accounting for background risk of TB, we found, as in adults, HIV was important risk factor for TB and that TB risk increases with immunosuppression. ART was strongly protective against TB, but took 2 years for full potential of protection against TB to be realised.

In adults, IRRs for TB given HIV around six have been estimated for populations with generalised HIV epidemics,^7^ corresponding to most newly diagnosed adult TB cases having HIV infection. Our IRR for children is comparable, but lower HIV prevalence is seen in children with TB due to a lower overall paediatric HIV prevalence. In adults, meta-analyses of cohorts of HIV patients suggest exponentially increasing IRR for TB with declining CD4 cell count,^9^ and current CD4 cell counts on ART strongly predict TB incidence.^81^ A systematic review and meta-analysis of the protective effect of ART against TB found a HR of 0.35 (0.28–0.44) across all baseline CD4 cell counts,^10^ comparable to our result for children. Long-term follow-up of adults on ART suggests 4–5 years for protection against TB to become fully established.^82^ Our analysis suggests that protection against TB in children on ART establishes more rapidly (1–2 years), consistent with faster CD4 reconstitution among children^83^ compared with adults.^84^ ^85^

Our findings have implications for patient care, guidelines, TB programmes and mathematical modelling. For clinicians, TB risk with HIV and ART informs patient treatment and appropriate family counselling. For TB programme officers, understanding risks informs resource allocation, and service configuration for HIV testing and treatment. These findings can inform evidence-based guideline development; timely given the recent revision of the WHO ART guidelines advocating universal treatment for children with HIV, irrespective of clinical and immunological stage. Finally, these data inform models of burden estimates and for evaluating the impact of interventions, in relation to HIV and ART.

Our review has limitations. Although our search strategy was comprehensive, studies were excluded if not representative of children with TB in that context. The cohorts identified were generally of low quality for our analyses, the most common shortcomings being around population representativeness and TB case-definitions. The majority of studies were from sub-Saharan Africa, where 90% of all HIV-infected children live, and the findings may not generalise to other regions. The studies spanned a long time period, and may not be representative of the current era. Statistical heterogeneity was high for summary statistics from both TB cohorts and especially HIV cohorts, reflecting diverse study settings and dates, and unreported clinical and methodological heterogeneity. However, measures of heterogeneity were comparable with those from similar studies.^86^ There were a number of limitations of our meta-analyses of TB cohort data. Controls were often not from the general population. This may partially explain the differences between HIV prevalence in study controls and UNAIDS paediatric HIV prevalence estimates. However, UNAIDS reports country-level estimates for children aged <15 years, which are not likely to reflect the HIV prevalence in younger children local to these studies. Difficulties in diagnosing TB in children with HIV may have led to differential detection of cases by HIV status, affecting the IRR from case-control TB cohorts. Moreover, estimates of the increased risk of TB progression will be confounded by higher exposure due to sharing households with HIV-infected parents, themselves at increased risk of TB. This makes it difficult to differentiate the direct biological impact of HIV from indirect effects. Implicitly, our analyses assume the same relationship between HIV, ART and TB holds regardless of population TB or HIV prevalence, which may not be the case across the very wide range of prevalences in the studies included.

A number of limitations apply to our meta-analyses of HIV data. CD4% categories were disparately reported, and we used category mid-points for analyses of CD4% and time-on-ART. Many studies allowed for unmasking of prevalent TB at ART initiation by excluding a certain period after initiation from comparisons, but this varied between studies. However, excluding all children diagnosed with TB soon after initiating ART may underestimate early incident TB in children who are still highly immunosuppressed. Only three HIV cohorts reported co-trimoxazole use,^14^ ^67^ ^80^ but as guidelines have varied over time and by setting, co-trimoxazole use may have been under-reported. Confounding by indication was not considered in estimates of ART protection (except for Edmonds *et al*^70^), and choice of covariates varied between studies. Our direct meta-analysis of the protective effect of ART was not able to include how long children had been on ART, and will average over the different distribution of treatment durations between studies.

We were not able to analyse age effects on TB, as data were seldom reported with multiple age stratifications. Our analysis of protection by time-on-ART may be confounded by age. Young children have particularly high TB progression risk, in part due to immature cell-mediated immune responses.^1^ The impact of age on efficacy of ART is also complex, as early ART initiation, at a better baseline immune status, leads to better immune reconstitution (although adherence can be challenging at the youngest ages due to a paucity of palatable formulations).^83^ ^87^ ^88^ The studies in our review were conducted over years when ART treatment guidelines, availability and formulations were evolving. The relevance of our findings to current universal treatment recommendations needs careful consideration; individual patient data meta-analysis would be highly informative on the impact of host characteristics on risk of TB, in particular the age of the child and age at which ART was initiated. As children are started on ART at higher CD4 values, the protective benefit of ART against TB may be lower.

Despite these limitations, internal consistency between results from different analyses increases the confidence in our conclusions. Our IRR from TB cohorts is comparable with the estimate of ‘severe’ compared with ‘not significant’ immune staging, recognising that HIV may confer an increased risk of TB even before immunosuppression is clinically evident. The mean IRR over a range of CD4% between 0% and 50% was 7.1 using the risk gradient point-estimate from our CD4 analysis—close to our IRR estimate for all HIV from TB cohorts. The mean protection against TB over the first 30 months on ART was 0.25—comparable to our meta-analysis of estimates of protection (although these analyses ultimately stem from the same data). The decline in TB rates by time-on-ART occurred on a similar timescale to the analysis presented by Li *et al*,^73^ although to a lower final value. Comparing our HIV and ART risk results suggests that children commencing ART are at higher risk of TB than HIV-uninfected children initially, but our results are too uncertain to specify whether long-term TB risks ART return to those of an HIV-uninfected child. For adults, TB risks established on long-term ART remain elevated over those of HIV-uninfected individuals.^82^

It is important to place ART in the context of other public health interventions. Our results suggest early diagnosis of infants and young children with HIV, followed by immediate ART, will reduce the pool of highly susceptible children and consequent progression to TB disease, providing further impetus to scale-up coverage among paediatric ART programmes. High HIV prevalence in the TB cohorts and high TB incidence in the HIV cohorts suggest that all children diagnosed with TB should be tested for HIV infection and all children with HIV should be regularly screened for TB disease. Scale-up of PMTCT will lead to fewer children becoming infected with HIV and is likely to be one of the most effective public health strategies to reduce paediatric HIV/TB; similarly, prompt diagnosis of TB in HIV-infected adults will reduce the risk of TB transmission to their HIV-infected children. TB control in adults more generally,^89^ and infection control measures, will reduce the risk of TB transmission to vulnerable children. IPT appears similarly effective at reducing TB incidence in HIV-infected children (around 70% reduction in incidence),^71^ ^80^ and evidence suggests that use in conjunction with ART is more protective than either intervention alone.^90^ Evidence for the benefit of co-trimoxazole in preventing incident TB in children is emerging;^14^ since it is now recommended long-term, co-trimoxazole prophylaxis may also help to reduce TB incidence among HIV-infected children on ART. Improving nutritional status among HIV-infected children may also be beneficial, as there is a high risk of TB in the context of malnutrition. Although individually these interventions are likely less effective than ART, their combination can be expected to protect the biggest number of children from developing TB.

## Conclusions

Our results indicate that HIV infection is a potent risk factor for TB in children, with a gradient of risk by indices of immunosuppression. ART is strongly protective against TB in children with HIV infection, taking up to 2 years for protection to become fully established, underscoring the importance of prompt ART initiation.
