# Supplementary material for: The impact of HIV and antiretroviral therapy on TB risk in children: a systematic review and meta-analysis
Source: Thorax. 2017 Jan 23;72(6):559–75. doi: 10.1136/thoraxjnl-2016-209421 (PMC5520282; doi:10.1136/thoraxjnl-2016-209421)
Supplement: supplementary material [file thoraxjnl-2016-209421supp001.pdf]

Supplementary Information:

The impact of HIV and antiretroviral therapy on tuberculosis risk in children: a systematic review and meta-analysis

P.J. Dodd[1], A.J. Prendergast[2,3], C. Beecroft[1], B. Kampmann[4,5] and J.A. Seddon[4]

1. School of Health and Related Research, University of Sheffield, Sheffield, UK
2. Blizard Institute, Queen Mary University of London, London, UK
3. Zvitambo Institute for Maternal and Child Health Research, Harare, Zimbabwe
4. Centre of International Child Health, Department of Paediatrics, Imperial College London, London, UK
5. Vaccines & Immunity Theme, MRC Unit The Gambia

Search strategy 3
Database searching 3
Table 1: OVID search strategy for MEDLINE and Embase 3
Reference searching 4
Citation searching 4
Table 2: Reviews for citation searching 4
Other sources 4
Descriptive epidemiology 5
TB cohorts 5
Figure 1: HIV prevalence in children with TB 5
HIV cohorts 5
Figure 2: TB incidence in children with HIV 6
Statistical analyses 7
TB cohorts 7
Relationship between odds ratios and incidence rate ratios 7
Case-control meta-analysis 7
Figure 3: Funnel plot for meta-analysis reported in main text. 8
Meta-analysis using UNAIDS HIV estimates 8
Figure 4: MCMC traceplots of 5 chains (TB cohorts with controls) 10
Figure 5: MCMC traceplots of 5 chains (TB cohorts without controls) 11
HIV cohorts 12
Data use by meta-analyses 12
Table 3: HIV cohort studies contributing data to each meta-analysis 12
Supplementary data file description 12

TB incidence by immunological stage ... 12
TB incidence by CD4 percentage ... 13
Figure 6: MCMC traceplots for Bayesian model of TB incidence by CD4% ... 15
TB incidence by time on ART ... 15
Protection from ART ... 16

Quality assessment ... 18

Newcastle-Ottawa Quality Assessment Scale for case-control studies: adaptation for TB cohorts ... 18

Newcastle-Ottawa Quality Assessment Scale for cohort studies: adaptation for HIV cohort analyses ... 19

Supplementary tables ... 22

Table 4: UNAIDS and population data ... 22
Table 6: Quality scores for TB cohorts (studies in grey included controls) ... 24
Table 7: Quality assessment for HIV cohorts: individual questions ... 26
Table 8: Quality assessment for HIV cohorts: score by analysis and domain ... 27

References ... 28

Search strategy

Database searching

Table 1: OVID search strategy for MEDLINE and Embase

#	Searches	Results
1	TB.ti,ab.	66,763
2	tuberculosis.ti,ab.	309,951
3	exp tuberculosis/	358,820
4	or/1-3	453,018
5	anti*retroviral.ti,ab.	92,404
6	ART.ti,ab.	121,074
7	ARV.ti,ab.	5,162
8	HAART.ti,ab.	24,787
9	exp Anti-HIV Agents/	174,914
10	or/5-9	332,619
11	child*.ti,ab.	2,314,958
12	p?ediatric.ti,ab.	494,285
13	neonat*.ti,ab.	451,007
14	new?born*.ti,ab.	286,653
15	infan*.ti,ab.	743,308
16	adolescen*.ti,ab.	417,922
17	prepubescen*.ti,ab.	1,661
18	("all infant (birth to 23 months)" or "all child (0 to 18 years)" or "newborn infant (birth to 1 month)" or "infant (1 to 23 months)" or "preschool child (2 to 5 years)" or "child (6 to 12 years)" or "adolescent (13 to 18 years)").mp. [mp=ti, ab, sh, hw, tn, ot, dm, mf, dv, kw, nm, kf, px, rx, ui]	50
19	or/11-18	3,635,854
20	HIV.ti,ab.	523,893
21	AIDS.ti,ab.	263,077
22	(HIV?1* or HIV?2*).ti,ab.	2,098
23	human immun*deficiency virus.ti,ab.	149,199
24	acquired immun*deficiency syndrome.ti,ab.	30,537
25	AIDS-Related Opportunistic Infections/	28,309
26	Acquired Immunodeficiency Syndrome/	198,936
27	exp HIV/	227,886
28	or/20-27	759,130
29	4 and 19 and (10 or 28)	4,994

Table 1 shows the complete OVID search strategy for MEDLINE and Embase and results as on 20/12/2014. (See also protocol)

Reference searching

We examined the 52 review or overview articles found by our database search[1-52] and examined the articles they listed in their bibliographies. This led to our considering 9 unique new abstracts, all of which were excluded.

Citation searching

The 5 relevant reviews found by our database search with the highest number of citations on Google Scholar were used (see Table 2). The citing articles listed by Google Scholar for these reviews were examined, yielding 87 unique new abstracts to screen. From these 39 full-text articles were examined and 3 additional articles contributed to the TB cohorts.[53-55]

Table 2: Reviews for citation searching

**Author**	**Year**	**Citations**
**Newton et al.[18]**	**2008**	**316**
**Marais et al.[22]**	**2007**	**161**
**Donald[36]**	**2002**	**117**
**Jeena et al.[56]**	**1996**	**61**
**Rekha et al.[13]**	**2007**	**50**

Other sources

A further HIV cohort analysis[57] (then under review) was brought to our attention after presenting preliminary results at the Union meeting in Cape Town, 2015.

Descriptive epidemiology

TB cohorts

A forest plot presenting the HIV prevalence observed in children with TB is shown in Figure 1.

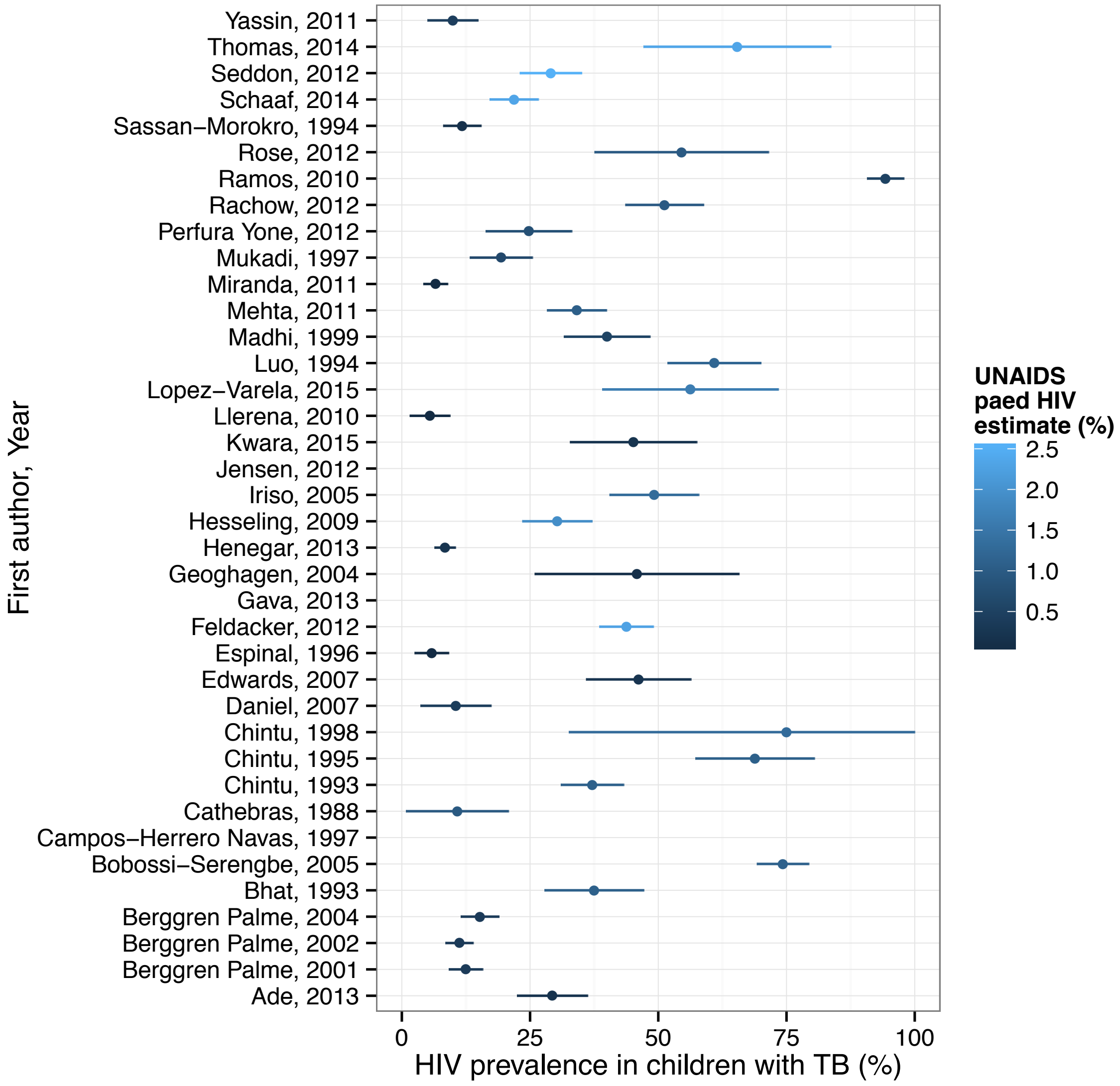

Figure 1: HIV prevalence in children with TB

HIV cohorts

A forest plot of TB incidence reported by cohorts of children with HIV is shown in Figure 2. Color codes study location and point shape the ART status of children.

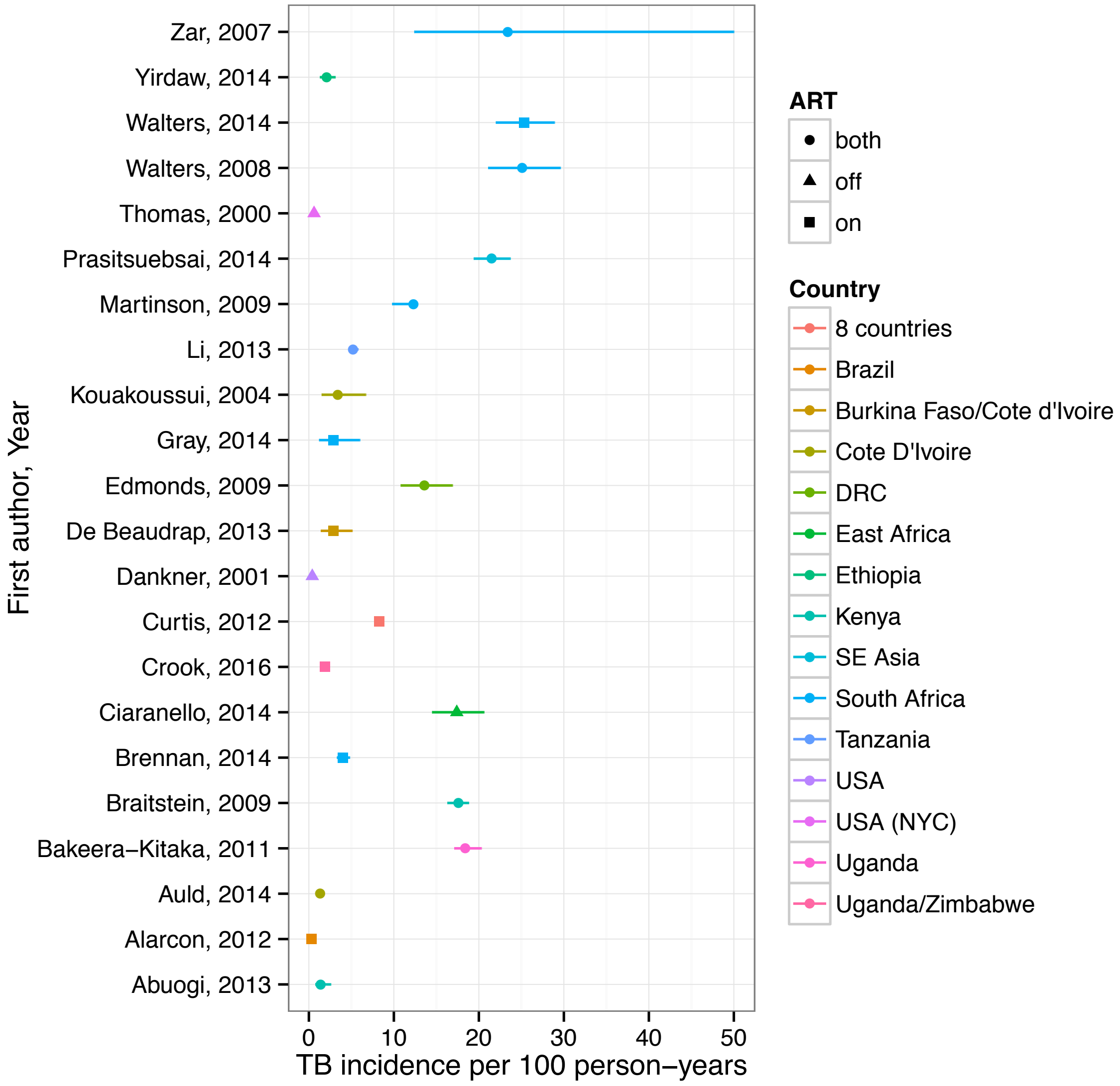

Figure 2: TB incidence in children with HIV

Statistical analyses

TB cohorts

Relationship between odds ratios and incidence rate ratios

Let *h* be the HIV prevalence in the general population, and *H* the HIV prevalence in TB cases. Let ρ be the incidence rate ratio (IRR) for developing TB disease if HIV infected. Let I_+ be the contribution to the incidence of TB due to HIV infected persons and I_- that due to HIV uninfected persons. By definition

$$\frac{I_+}{h} = \rho \frac{I_-}{(1-h)}$$

The OR for HIV given TB is

$$OR = \frac{H/(1-H)}{h/(1-h)} = \frac{I_+/I_-}{h/(1-h)} = \rho$$

Case-control meta-analysis

This analysis used the metafor package for R.[58] Funnel plots for the meta-analysis of TB cohorts reported in the main text as presented in Figure 3.

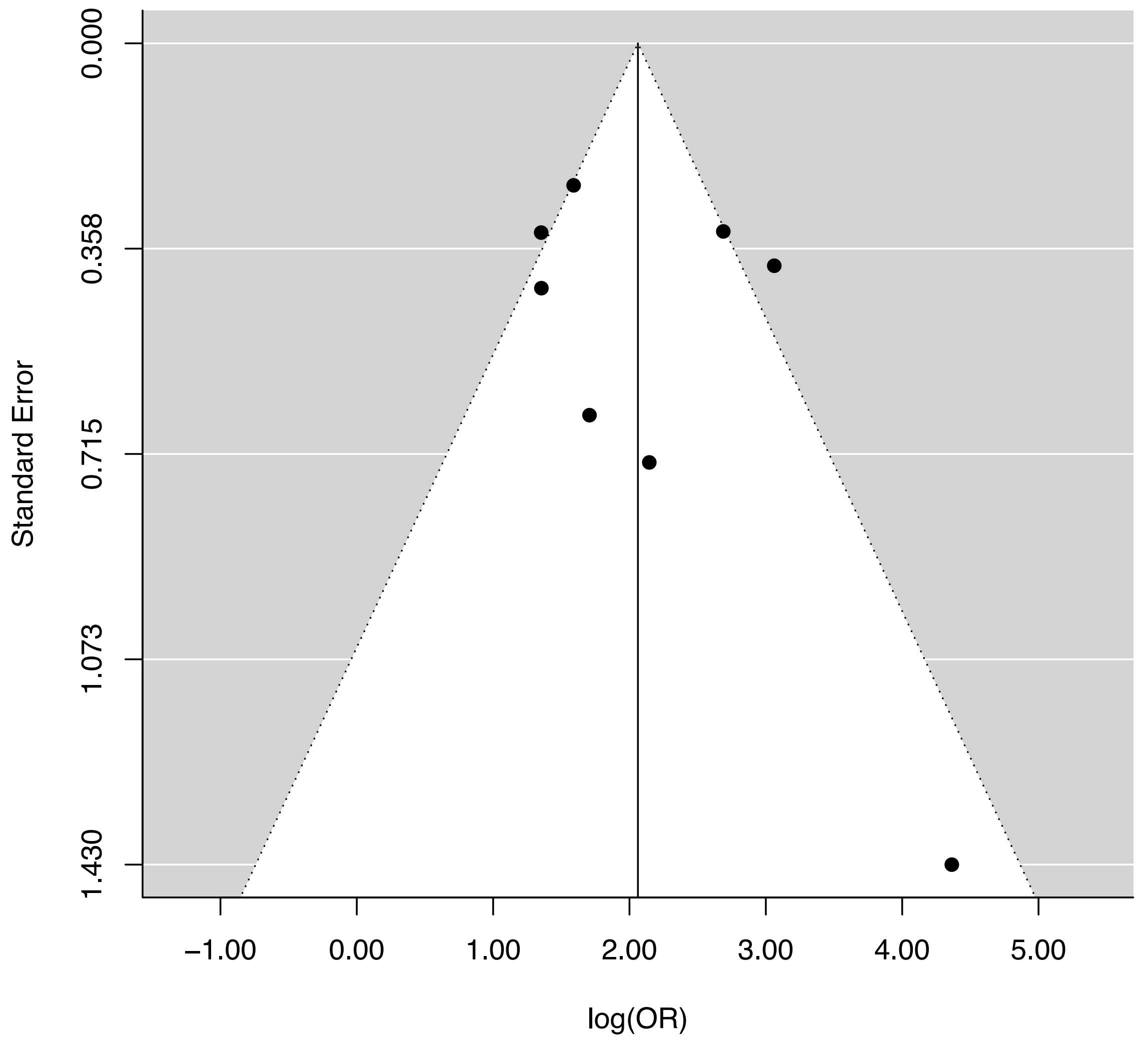

Figure 3: Funnel plot for meta-analysis reported in main text.

Meta-analysis using UNAIDS HIV estimates

For studies where HIV prevalence in controls without TB were not available, we sought national UNAIDS estimates of HIV prevalence in children aged 0-15. This data was available for all of the 8 studies with their own controls and a further 27 studies without their own controls (see Table 4). The UNAIDS HIV prevalence mid-point estimates and 95% uncertainty ranges were interpreted as equivalent numerator (*n*) and denominators (*N*) using standard binomial confidence interval formulae.

We undertook a Bayesian meta-analysis of these data where the relationship between the HIV prevalence estimated nationally by UNAIDS and the locally appropriate HIV prevalence for controls was informed by the studies where both control HIV prevalence and UNAIDS HIV prevalence were available.

The hierarchical model used was therefore

$$n_i^{TB} \sim Bin(N_i^{TB}, p_i^{TB})$$
$$n_i^{C} \sim Bin(N_i^{C}, p_i^{C})$$
$$n_i^{U} \sim Bin(N_i^{U}, p_i^{U})$$
$$logit(p_i^{TB}) = \mu_i + \delta_i + \eta_i$$
$$logit(p_i^{C}) = \mu_i + \eta_i$$
$$logit(p_i^{U}) = \mu_i$$
$$\delta_i \sim N(m_\delta, s_\delta^2)$$
$$\eta_i \sim N(m_\eta, s_\eta^2)$$
$$\mu_i \sim N(m_\mu, s_\mu^2)$$
$$m_x \sim N(1, 10^3)$$
$$s_x^2 \sim IG(5{,}25)$$
$$x \in \{\delta, \eta, \mu\}$$

where *IG* is the inverse gamma distribution. The distribution $N(m_\eta, s_\eta^2)$ therefore characterizes the typical relationship between UNAIDS national HIV prevalence estimates, and $\delta_i \sim N(m_\delta, s_\delta^2)$ characterizes the effect of HIV on TB risk, analogously to the random effects model in the main paper.

Gibbs sampling implemented in the R statistical programming environment[59] was used (together with a normal approximation to the likelihood) to perform Markov chain Monte Carlo (MCMC) sampling from the posteriors of all the study-specific 'random effects' parameters (e.g. δ_i), and the parameters at the population-of-studies level of the hierarchy (e.g. *ml*). Chains were run for 5,000 iterations, discarding the first 1,000 as burn in, and thinning to every 1 in 10 to reduce autocorrelation. Five chains with random starting points for the μ_i parameters are shown in Figure 4 and Figure 5. The upper confidence intervals of the Gelman-Rubin statistics were ≤ 1.2 for all study-specific parameters.

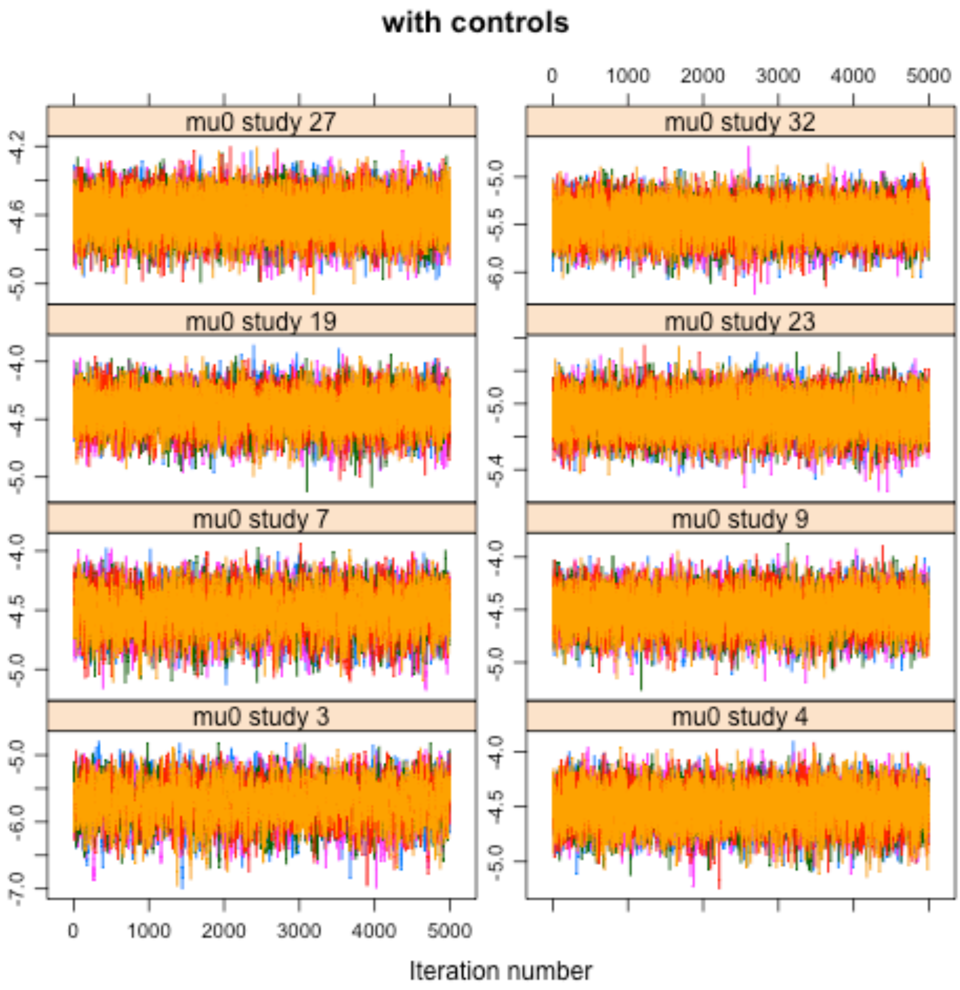

Figure 4: MCMC traceplots of 5 chains (TB cohorts with controls)

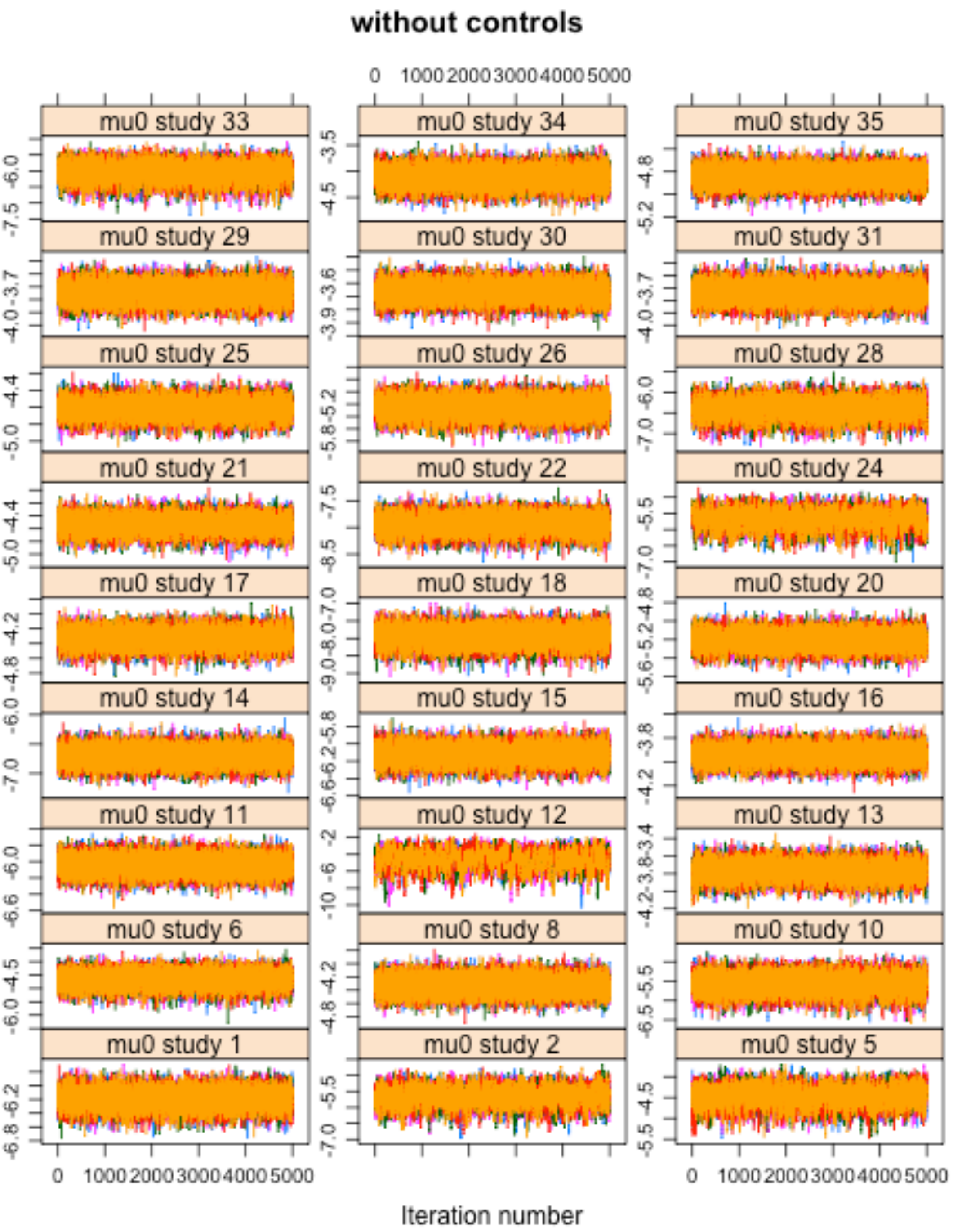

Figure 5: MCMC traceplots of 5 chains (TB cohorts without controls)

The posterior mean (95% credible interval [CrI]) for the OR of TB given HIV was 7.0 (95%CrI: 5.7 – 8.5) and the estimated OR relating control HIV prevalence to UNAIDS estimates was 7.3 (95%CrI: 5.9 – 8.8). The posteriors for $m_{\delta}$and m_{η} exhibited a high degree of correlation (-0.83). A forest plot comparing this Bayesian analysis with a Bayesian meta-analysis analogous to the above but using only the case-control studies controls is shown in the main article. The Bayesian analysis described above additionally estimates the effect sizes for studies without their own controls.

HIV cohorts

Data use by meta-analyses

Table 3: HIV cohort studies contributing data to each meta-analysis

STUDY	Meta-analysis			
	Clinical staging	CD4%	ART time	ART HR
Abuogi, 2013	X			
Auld, 2014		X	X	
Bakeera-Kitaka, 2011			X	X
Braitstein, 2009			X	X
Brennan, 2014		X	X	
Ciaranello, 2014		X		
Crook, 2016		X	X	
Curtis, 2012			X	
Dankne, 2001		X		
De Beaudrap, 2013		X	X	
Edmonds, 2009	X		X	X
Kouakoussui, 2004		X		
Li, 2013	X		X	X
Martinson, 2009				X
Walters, 2014			X	
Yirdaw,2014				X

Supplementary data file description

The supplementary data file contains TB incidence per 100 person-years (with 95% confidence intervals where appropriate) reported by HIV cohort studies, stratified by: time on ART, clinical immunosuppression staging, and CD4 percentage category.

TB incidence by immunological stage

Three studies reported TB incidence stratified by WHO immunological staging (Not significant, Mild, Advanced, Severe), reporting incidence relative to the base category (Not significant). These data are graphed in the main article. We performed a random effects meta-

analysis using the metafor package in R[58], treating each stage as a separate stratum. The pooled relative incidence is shown in the main article figure by a purple dashed line, points and error-bars. The pooled relative incidence in the Severe category was 5.0 (95%: 4.0 – 6.0) with I-squared heterogeneity statistic I^2 = 87.1%.

TB incidence by CD4 percentage

Six studies reported TB incidence reported TB incidence stratified by more than one CD4% category, each study using a different set of CD4% categories (see Supplementary data file). Additionally, one study[60] analysed a large cohort to produce an estimate of the factor change, *F*, in TB incidence associated with a unit increase in CD4 percentage, obtaining 0.94 (95%CI: 0.91 – 0.97).

To produce a pooled estimate of the gradient in the logarithmic TB incidence rate ratio with respect to CD4% change, α, we used a Bayesian model where the TB incidence I_{ij} for the *i*-th study (i=1,...,6) at the j-th CD4% category mid-point c_j follows:

$$\log\,(I_{ij}) \sim N(\alpha_i . c_{ij} + \beta_i, s_{ij}^2)$$
$$\alpha_i \sim N(m_\alpha, s_\alpha^2)$$
$$\beta_i \sim N(m_\beta, s_\beta^2)$$
$$m_x \sim N(M_x, S_x^2)$$
$$S_x^2 \sim IG(a, b)$$
$$x \in \{\alpha, \beta\}$$

with the β_i capturing the overall level of TB incidence in each study, and where s_{ij}^2 are derived from the reported confidence interval for each incidence. Working with within-study-differenced data (and dropping points with zero incidence from the analysis) means we can ignore β_i:

$$\log\big(I_{ij}\big) - \log(I_{i0}) \sim N\big(\alpha_i . (c_{ij} - c_{i0}), s_{ij}^2 + s_{i0}^2\big)$$
$$\alpha_i \sim N(m_\alpha, s_\alpha^2)$$
$$m_\alpha \sim N(M_\alpha, S_\alpha^2)$$
$$S_\alpha^2 \sim IG(a, b)$$

We also introduce an additional term in the data log-likelihood describing the factor, *F*, from Crook et al.,[57] which is assumed to be normally distributed on a log scale with variance s_0^2 derived from the reported confidence intervals:

$$\alpha_0 \sim N(\log(F)\,, s_0^2)$$

This means that (for $i \geq 1$):

$$\alpha_i | m_\alpha, s_\alpha^2, \mu_i, \sigma_i^2 \sim N(\mu_i, \sigma_i^2)$$

where

$$\mu_i = \sigma_i^2 \left(\sum\nolimits_j \frac{\delta I_{ij} . \delta c_{ij}}{s_{ij}^2 + s_{i0}^2} + \frac{m_\alpha}{s_\alpha^2} \right)$$

$$\sigma_i^2 = \left(\sum\nolimits_j \frac{\delta c_{ij}^2}{s_{ij}^2 + s_{i0}^2} + \frac{1}{s_\alpha^2} \right)^{-1}$$

(with $\delta x_{ij} = x_{ij} - x_{i0}$); and for $i = 0$,

$$\alpha_0 | m_\alpha, s_\alpha^2 \sim N\left(\frac{\frac{\log(F)}{s_0^2} + \frac{m_\alpha}{s_\alpha^2}}{(s_0^{-2} + s_\alpha^{-2})}, (s_0^{-2} + s_\alpha^{-2})^{-1} \right)$$

Gibbs sampling can then be used since:

$$m_\alpha | \{\alpha_i\}, s_\alpha^2, M_\alpha, S_\alpha^2 \sim N\left(\frac{\frac{\sum_i \alpha_i}{s_\alpha^2} + \frac{M_\alpha}{S_\alpha^2}}{(S_\alpha^{-2} + n s_\alpha^{-2})}, (S_\alpha^{-2} + n s_\alpha^{-2})^{-1} \right)$$

(where *n* is the number of *i*'s including 0), and

$$s_\alpha^2 | \{\alpha_i\}, m_\alpha, M_\alpha, S_\alpha^2 \sim IG\left(a + \frac{n}{2}, b + \frac{\sum_i (m_\alpha - \alpha_i)^2}{2} \right)$$

This scheme was implemented in R. Parameters *a* and *b* were chosen to correspond to a prior for s_α^2 with a mean of 0.1 and a variance of 10. Chains were run for 5,000 iterations, discarding the first 1,000 as burn in, and thinning to every 1 in 5 to reduce autocorrelation. Five chains with random starting points (see Figure 6); the upper confidence intervals of the Gelman-Rubin statistics were ≤ 1.0 (within rounding errors) for all parameters.

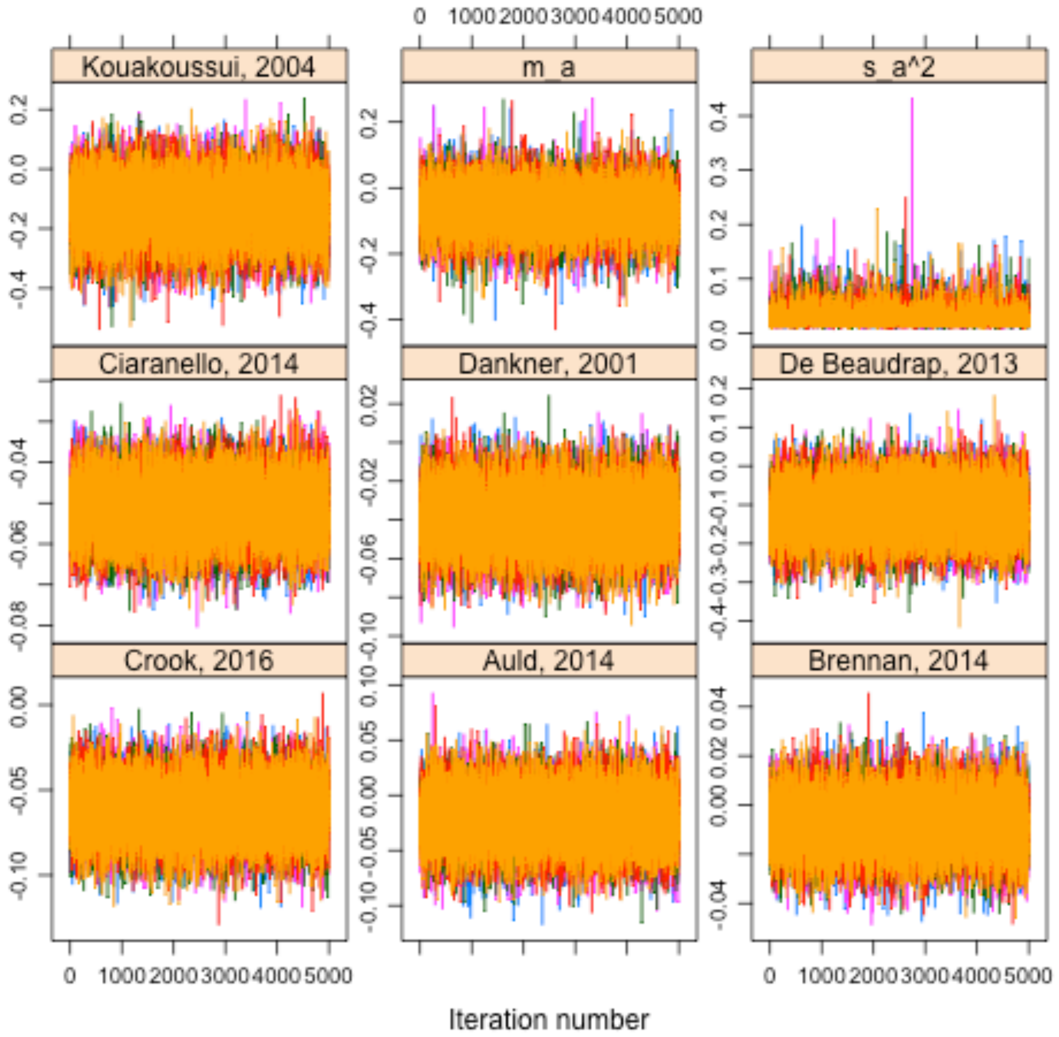

Figure 6: MCMC traceplots for Bayesian model of TB incidence by CD4%

The pooled estimate was m_α = -0.063 (95%CrI: -0.188 – +0.063). The forest plot of estimates for each study are shown in the main article.

TB incidence by time on ART

A figure the main article shows the data on TB incidence by months-since-ART-initiation reported by 9 studies (colored lines, points and error-bars). The data have been aligned for this plot to standardize for overall TB incidence by shifting a study-specific time-series up or down (on the log-scale) so that its first point lies at the value predicted by a linear model fitted to all preceding (i.e. leftward) adjusted data. This allows visualization of the relative TB incidence by time-on-ART by scaling the data each study so that it is in line with the rest.

A non-linear mixed effects regression model was fitted using the lme4 package in R.[61] The original data set was replicated 10 times, with the log incidences perturbed by a Gaussian random noise with variances chosen to reproduce the reported confidence intervals for each

point; thus allowing an approximation to the data likelihood. For the i-th study, the TB incidence I_{it} at time *t* is taken to follow:

$$\log(I_{it}) = c_i + b_i.(1 - \exp(-a_i.t))$$

where a_i, $b_i$and c_i are treated as random effects, and determine the early rate of change and asymptotic level of protection, respectively. The study-specific intercepts c_i set the overall level of TB incidence in each study and are not of interest to us.

The figure the main article also shows the dashed black curve corresponding to the pooled estimated trajectory with 95% uncertainty estimates in gray (constructed from 1,000 sample trajectories based on normal samples using the variance-covariance matrix). The estimated asymptotic protection was *HR* = 0.10 (95%CI: 0.04 - 0.25) and the saturation timescale corresponded to 4.5 months.

Protection from ART

Six studies reported a hazard ratio for the protective effect of ART against TB incidence (see

Table 5). In addition, Abuogi et al.[62] estimated a factor change in TB incidence of 0.91 (95%: 0.86 – 0.95) per month on ART. We undertook a random-effects meta-analysis of these studies (see forest plot in the main article). The pooled hazard ratio for TB on ART was 0.30 (95%CI: 0.21 - 0.39). The I-squared heterogeneity statistic was I^2 = 79%.

Quality assessment

Quality was assessed by a modified version of the Newcastle-Ottawa scale: the case-control instrument for the TB cohorts and the cohort instrument for HIV cohorts. Five version of the instrument were applied to HIV cohorts to generate a separate quality for each analysis. Not all questions were relevant to each version.

Newcastle-Ottawa Quality Assessment Scale for case-control studies: adaptation for TB cohorts

Note: A study can be awarded a maximum of one star for each numbered item within the Selection and Exposure categories. A maximum of two stars can be given for Comparability.

This assessment was used to evaluate studies that measured the proportion of cases (children who had TB disease) with HIV and the proportion of controls (children without TB disease) with HIV.

Selection

1) Is the case definition of TB adequate?
   - a) Yes, using carefully described criteria ⍟
   - b) Yes, based on clinician treatment decision
   - c) No description

2) Representativeness of the children with TB in the study as compared to children with TB in the communities that they were drawn from
   - a) Consecutive cases or obviously representative series of cases ⍟
   - b) Potential for selection biases or not stated

3) Selection of controls (children without TB)
   - a) Community controls ⍟
   - b) Hospital controls
   - c) No description of where controls drawn from

4) Exclusion of TB disease in controls
   - a) Controls documented to not have TB disease ⍟
   - b) No description of TB status

Comparability

1) Comparability
   - a) The study controlled for age of child when matching cases and controls ⍟
   - b) The study controlled for additional factors such as gender, BCG vaccination status, nutrition status, socio-economic status ⍟

Exposure

1) Ascertainment of HIV status
   a) Laboratory record ⍟
   b) Interview of patient blinded to case/control status ⍟
   c) Interview not blinded to case/control status
   d) Written self-report or medical record only
   e) No description

2) Was the same method of ascertainment of HIV status made for cases and controls?
   a) Yes ⍟
   b) No

3) Was the proportion tested for HIV?
   a) The same in both groups ⍟
   b) Details of those not tested are described
   c) The proportion tested was different between the cases and controls or not documented

Newcastle-Ottawa Quality Assessment Scale for cohort studies: adaptation for HIV cohort analyses

Note: A study can be awarded a maximum of one star for each numbered item within the Selection and Outcome categories. A maximum of two stars can be given for Comparability

We regarded the HIV cohorts to being pertinent to 5 analyses. The same studies could be regarded as having different quality for the purposes of these distinct analyses. The analyses were:

A. TB incidence in children with HIV. All studies included a reported TB incidence that could be taken as a measure of TB incidence in children with TB. Comparability/control questions were not applicable.
B. TB incidence by clinical immunosuppression grade. Three studies were used to estimate the effect of clinical immune grade on TB incidence. This analysis involved within-cohort comparisons and so control questions were not applicable.
C. TB incidence by CD4 percentage. Seven studies were used to inform an analysis of the effect of CD4% on TB incidence. This analysis involved within-cohort comparisons and so control questions were not applicable.
D. TB incidence by time on ART. Ten studies were used to estimate the effect of time-on-ART on TB incidence. This analysis involved within-cohort comparisons and so control questions were not applicable.
E. The effect of ART on TB incidence. Six studies were used to estimate the protective effect against TB incidence of ART.

Some questions (e.g. surrounding outcomes) were the same for all 5 analyses. Other questions were modified by addition of text specific to the analysis. This is represented below in square brackets: *[incidence/immunosuppression/CD4%/time-on-ART/ART]=[A/B/C/D/E].*

Selection

S1) Representativeness of the children with HIV *[*/and a given level of immunosuppression/and a given CD4%/who had been on ART for a certain time/and not on ART]*

a) Truly representative of the average child with HIV *[/and a given level of immunosuppression/and a given CD4%/who had been on ART for a certain time/and not on ART]*
in the community ⍟
b) Somewhat representative of the average child with HIV *[/and a given level of immunosuppression/and a given CD4%/who had been on ART for a certain time/and not on ART]* in the community ⍟
c) Selected group of children e.g. those admitted to hospital, being seen in clinic etc.
d) No description of the representativeness of the cohort

S2) Selection of children on ART *[ART ONLY – rest are cross-cohort comparisons and obtain no star]*
a) Drawn from the same community as the children not on ART in time and place ⍟
b) Drawn from a different source in time and/or place
c) No description of the derivation of the children

S3) Ascertainment of HIV status
a) Result taken from laboratory records ⍟
b) Result obtained from medical records or patient/parent interview ⍟
c) No description

S4) Demonstration that the child did not have TB at the start of the study
a) Yes ⍟
b) No

Comparability†

C1) Comparability of cohorts on the basis of the design or analysis
a) The study controls for age of child when comparing different *[/levels of immunosuppression/ CD4%/times on ART/ART status]* ⍟
b) Study controls for other factors such as gender, socioeconomic status, BCG vaccination status, nutritional status, etc.‡ ⍟

Outcome

O1) Assessment of incident TB disease
a) Microbiologically confirmed TB disease ⍟
b) Clinically diagnosed TB disease using defined criteria ⍟
c) Clinician decision to treat for TB disease
d) No description made

O2) Was follow-up long enough for outcomes to occur
a) Yes, follow-up was for over a year after recruitment in the majority of children ⍟

---

* The blank space indicates no text added.
† This section does not apply to the incidence analysis (A)
‡ Including level of immunosuppression except for the immunosuppression (B) and CD4% (C) analyses

b) No, follow-up often short or not described

O3) Adequacy of follow up of cohorts

a) Complete follow up - all subjects accounted for ⊛

b) Subjects lost to follow up unlikely to introduce bias - small number lost to follow-up (<20%), or description provided of those lost ⊛

c) Loss to follow-up >20% and no description of those lost

d) No statement

Supplementary tables

Table 4: UNAIDS and population data

First Author, Year	UNAIDS HIV estimate (numbers, age 0-15)	UN population estimate (millions, age 0-15)
Ade, 2013[63]	7900 [6800-9000]	3.867
Berggren Palme, 2004[64]	97000 [76000-130000]	27.09
Berggren Palme, 2001[65]	97000 [76000-130000]	27.09
Bhat, 1993[66]	40000 [34000-47000]	3.695
Bobossi-Serengbe, 2005[67]	17000 [14000-22000]	1.534
Cathebras, 1998[68]	14000 [11000-19000]	1.479
Chintu, 1993[69]	40000 [34000-47000]	3.695
Chintu, 1998[70]	50000 [44000-57000]	3.862
Chintu, 1995[71]	40000 [34000-47000]	3.695
Daniel, 2007[72]	210000 [150000-250000]	54.64
Edwards, 2007[73]	61000 [55000-68000]	25.89
Espinal, 1996[74]	1400 [1000-21000]	2.861
Feldacker, 2012[75]	150000 [130000-160000]	6.626
Gava, 2013[76]	14000 [11000-19000]	51.53
Geoghagen, 2004[77]	1000 [800-1100]	0.841121
Henegar, 2013[78]	63000 [57000-70000]	28.69
Hesseling, 2009[79]	290000 [270000-310000]	15.15
Iriso, 2005[80]	170000 [150000-190000]	13.01
Llerena, 2010[81]	4600 [3500-5900]	13.24
Luo, 1994[82]	45000 [39000-52000]	3.781
Madhi, 1999[83]	82000 [75000-90000]	15.06
Mehta, 2011[84]	190000 [170000-220000]	17.79
Miranda, 2011[85]	18000 [15000-21000]	51.3
Mukadi, 1997[86]	48000 [44000-54000]	7.527
Berggren Palme, 2002[87]	97000 [76000-130000]	27.09
Rachow, 2012[88]	190000 [170000-210000]	19.44
Ramos, 2010[89]	17000 [14000-19000]	33.78
Rose, 2012[90]	190000 [170000-210000]	19.44
Sassan-Morokro, 1994[91]	9300 [7700-12000]	5.649
Schaaf, 2014[92]	350000 [330000-380000]	15.11
Seddon, 2012[93]	380000 [350000-400000]	15.18
Thomas, 2014[94]	350000 [330000-380000]	15.11
Yassin, 2011[95]	150000 [130000-180000]	34.23
Kwara, 2015[54]	22000 [17000-30000]	9.942
Lopez-Varela, 2015[53]	170000 [150000-210000]	10.51
Perfura Yone, 2012[55]	59000 [54000-63000]	7.575

Table 5: HIV cohorts quality and ART protection

First author, year	Hazard ratio for TB on ART
Bakeera-Kitaka, 2011[96]	0.41 (0.30-0.54)
Braitstein, 2009[97]	0.15 (0.12-0.20)
Edmonds, 2009[98]	0.51 (0.27-0.94)
Li, 2013[99]	0.30 (0.20-0.40)
Martinson, 2009[100]	0.30 (0.20-0.40)
Yirdaw, 2014[101]	0.32 (0.24-0.43)

Table 6: Quality scores for TB cohorts (studies in grey included controls)

	Selection				Comparability	Exposure			Total
**Question**	1	2	3	4	1	1	2	3	
**Max score**	⍟	⍟	⍟	⍟	⍟⍟	⍟	⍟	⍟	**9 (3)§**
Ade, 2013	0	1	NA	NA	NA	0	NA	NA	1
Berggren Palme, 2004	1	1	NA	NA	NA	1	NA	NA	3
Berggren Palme, 2001	0	1	0	1	1	1	1	1	7
Bhat, 1993	1	1	0	1	0	1	1	1	7
Bobossi-Serengbe, 2005	0	1	NA	NA	NA	1	NA	NA	2
Campos-Herrero Navas, 1997	1	1	NA	NA	NA	0	NA	NA	2
Cathebras, 1998	1	1	NA	NA	NA	1	NA	NA	2
Chintu, 1993	1	1	0	0	0	1	1	1	5
Chintu, 1998	0	0	NA	NA	NA	1	NA	NA	1
Chintu, 1995	1	1	0	0	0	1	1	1	5
Daniel, 2007	1	1	NA	NA	NA	1	NA	NA	3
Edwards, 2007	1	1	NA	NA	NA	1	NA	NA	3
Espinal, 1996	1	1	NA	NA	NA	1	NA	NA	3
Feldacker, 2012	0	1	NA	NA	NA	0	NA	NA	1
Gava, 2013	0	1	NA	NA	NA	1	NA	NA	2
Geoghagen, 2004	0	1	NA	NA	NA	1	NA	NA	2
Henegar, 2013	0	1	NA	NA	NA	0	NA	NA	1
Hesseling, 2009	1	0	NA	NA	NA	1	NA	NA	2
Hussain, 2007	0	1	NA	NA	NA	1	NA	NA	2
Iriso, 2005	1	1	NA	NA	NA	1	NA	NA	3
[illegible]									

Llerena, 2010	1	0	NA	NA	NA	1	NA	NA	2
Luo, 1994	1	1	0	0	0	1	1	1	5
Madhi, 1999	0	1	NA	NA	NA	1	NA	NA	2
Mehta, 2011	1	1	NA	NA	NA	1	NA	NA	3
Miranda, 2011	0	0	NA	NA	NA	0	NA	NA	0
Mukadi, 1997	1	1	1	1	1	1	1	1	8
Berggren Palme, 2002	1	1	NA	NA	NA	1	NA	NA	3
Panigatti, 2014	0	1	NA	NA	NA	0	NA	NA	1
Rachow, 2012	1	0	NA	NA	NA	1	NA	NA	2
Ramos, 2010	0	1	NA	NA	NA	0	NA	NA	1
Rose, 2012	1	1	0	1	0	1	1	1	6
Sassan-Morokro, 1994	1	1	NA	NA	NA	1	NA	NA	3
Schaaf, 2014	1	0	NA	NA	NA	1	NA	NA	2
Seddon, 2012	1	0	NA	NA	NA	1	NA	NA	2
Shahab, 2004	0	1	NA	NA	NA	1	NA	NA	2
Thomas, 2014	1	1	NA	NA	NA	1	NA	NA	3
Yassin, 2011	1	1	1	1	0	1	1	0	6
Kwara, 2015	0	0	NA	NA	NA	0	NA	NA	0
Lopez-Varela, 2015	1	1	NA	NA	NA	1	NA	NA	3
Perfura Yone, 2012	1	1	NA	NA	NA	1	NA	NA	3

Table 7: Quality assessment for HIV cohorts: individual questions

**Analysis**	(all)					A	B		C		D		E		
**Question**	S3	S4	O1	O2	O3	S1	S1	C1	S1	C1	S1	C1	S1	S2	C1
**Max score**	⍟	⍟	⍟	⍟	⍟	⍟	⍟	⍟⍟	⍟	⍟⍟	⍟	⍟⍟	⍟	⍟	⍟⍟
Abuogi, 2013	1	1	1	1	1	0	0	0	NA	NA	NA	NA	NA	NA	NA
Alarcon, 2012	1	1	0	1	1	0	NA	NA	NA	NA	NA	NA	NA	NA	NA
Auld, 2014	1	0	0	1	0	0	NA	NA	0	2	1	0	NA	NA	NA
Bakeera-Kitaka, 2011	1	0	0	0	1	0	NA	NA	NA	NA	1	0	0	1	2
Braitstein, 2009	1	0	1	0	0	0	NA	NA	NA	NA	1	0	0	1	2
Brennan, 2014	1	0	0	0	0	0	NA	NA	0	2	1	2	NA	NA	NA
Ciaranello, 2014	1	0	0	0	1	0	NA	NA	0	0	NA	NA	NA	NA	NA
Crook, 2016	1	1	0	1	1	0	NA	NA	0	2	1	0	NA	NA	NA
Curtis, 2012	1	0	0	1	0	0	NA	NA	NA	NA	0	0	NA	NA	NA
Dankne, 2001	1	0	0	1	0	0	NA	NA	0	0	NA	NA	NA	NA	NA
De Beaudrap, 2013	1	0	1	1	1	0	NA	NA	0	0	1	0	NA	NA	NA
Edmonds, 2009	1	1	0	1	1	0	0	2	NA	NA	1	0	0	1	2
Gray, 2014	1	1	1	0	1	0	NA	NA	NA	NA	NA	NA	NA	NA	NA
Kouakoussui, 2004	1	0	0	1	0	0	NA	NA	0	0	NA	NA	NA	NA	NA
Li, 2013	1	1	0	0	0	0	0	2	NA	NA	0	2	0	1	2
Martinson, 2009	1	1	0	1	0	0	NA	NA	NA	NA	NA	NA	0	1	2
Prasitsuebsai, 2014	1	0	0	1	0	0	NA	NA	NA	NA	NA	NA	NA	NA	NA
Thomas, 2000	1	0	0	1	1	1	NA	NA	NA	NA	NA	NA	NA	NA	NA
Walters, 2008	1	0	1	1	1	0	NA	NA	NA	NA	NA	NA	NA	NA	NA
Walters, 2014	1	1	0	0	0	0	NA	NA	NA	NA	1	0	NA	NA	NA
Yirdaw,2014	1	1	0	1	0	0	NA	NA	NA	NA	NA	NA	0	1	2
Zar, 2007	1	0	1	0	0	0	NA	NA	NA	NA	NA	NA	NA	NA	NA

Table 8: Quality assessment for HIV cohorts: score by analysis and domain

Analysis	Incidence				Immune staging				CD4 percentage				Time-on-ART				ART use			
**Domain**	Selection	Comparability	Outcomes	**Total**	Selection	Comparability	Outcomes	**Total**	Selection	Comparability	Outcomes	**Total**	Selection	Comparability	Outcomes	**Total**	Selection	Comparability	Outcomes	**Total**
**Max score**	3	NA	3	**6**	3	2	3	**8**	3	2	3	**8**	3	2	3	**8**	4	2	3	**9**
Abuogi, 2013	2	NA	3	**5**	2	0	3	**5**												
Alarcon, 2012	2	NA	2	**4**																
Auld, 2014	1	NA	1	**2**					1	2	1	**4**	2	0	1	**3**				
Bakeera-Kitaka, 2011	1	NA	1	**2**									2	0	1	**3**	2	2	1	**5**
Braitstein, 2009	1	NA	1	**2**									2	0	1	**3**	2	2	1	**5**
Brennan, 2014	1	NA	0	**1**					1	2	0	**3**	2	2	0	**4**				
Ciaranello, 2014	1	NA	1	**2**					1	0	1	**2**								
Crook, 2016	2	NA	2	**4**					2	2	2	**6**	3	0	2	**5**				
Curtis, 2012	1	NA	1	**2**									2	0	1	**4**				
Dankne, 2001	1	NA	1	**2**					1	0	1	**2**								
De Beaudrap, 2013	1	NA	3	**4**					1	0	3	**4**	2	0	3	**5**				
Edmonds, 2009	2	NA	2	**4**	2	0	2	**4**					3	0	2	**5**	3	2	2	**7**
Gray, 2014	3	NA	1	**4**																
Kouakoussui, 2004	1	NA	1	**2**					1	0	1	**2**								
Li, 2013	2	NA	0	**2**	2	2	0	**4**					3	2	0	**5**	3	2	0	**5**
Martinson, 2009	2	NA	1	**3**													3	2	1	**6**
Prasitsuebsai, 2014	1	NA	1	**2**																
Thomas, 2000	2	NA	2	**4**																
Walters, 2008	1	NA	3	**4**																
Walters, 2014	2	NA	0	**2**									3	0	0	**3**				
Yirdaw,2014	2	NA	1	**3**													3	2	1	**6**
Zar, 2007	1	NA	1	**2**																

References

1. Zar HJ. Chronic lung disease in human immunodeficiency virus (HIV) in children. *Pediatric pulmonology* 2008; **43**(1): 1-10.
2. Zar HJ. Global paediatric pulmonology: out of Africa. *Paediatric respira* 2006; **7 Suppl 1**: S226-8.
3. Zar HJ. Pneumonia in HIV-infected and HIV-uninfected children in deve countries: epidemiology, clinical features, and management. *Current opinion in* *medicine* 2004; **10**(3): 176-82.
4. Wood R. When to start antiretroviral therapy in children with TB? *Exper* *anti-infective therapy* 2010; **8**(10): 1101-4.
5. Whittaker E, Kampmann B. Perinatal tuberculosis: new challenges in the and treatment of tuberculosis in infants and the newborn. *Early human developm* **84**(12): 795-9.
6. Walls T, Shingadia D. Global epidemiology of paediatric tuberculosis. *Th* *infection* 2004; **48**(1): 13-22.
7. Verhagen LM, Warris A, van Soolingen D, de Groot R, Hermans PWM. immunodeficiency virus and tuberculosis coinfection in children: challenges in d treatment. *The Pediatric infectious disease journal* 2010; **29**(10): e63-70.
8. Venturini E, Turkova A, Chiappini E, Galli L, de Martino M, Thorne C. and HIV co-infection in children. *BMC infectious diseases* 2014; **14 Suppl 1**: S5
9. Van Dyke RB. Opportunistic infections in pediatric HIV disease. *Annals* *York Academy of Sciences* 1993; **693**: 158-65.
10. Swaminathan S, Rekha B. Pediatric tuberculosis: global overview and ch *Clinical infectious diseases : an official publication of the Infectious Diseases So* *America* 2010; **50 Suppl 3**: S184-94.
11. Swaminathan S. Tuberculosis in HIV-infected children. *Paediatric respi* *reviews* 2004; **5**(3): 225-30.
12. Schaaf HS, Collins A, Bekker A, Davies PDO. Tuberculosis at extremes *Respirology (Carlton, Vic)* 2010; **15**(5): 747-63.
13. Rekha B, Swaminathan S. Childhood tuberculosis - global epidemiology impact of HIV. *Paediatric respiratory reviews* 2007; **8**(2): 99-106.
14. Poerksen G, Kazembe P, Graham S. Challenges of Childhood TB/HIV M in Malawi. *Malawi medical journal : the journal of Medical Association of Mala* **19**(4): 142-8.
15. Pillay T, Khan M, Moodley J, Adhikari M, Coovadia H. Perinatal tuberc HIV-1: considerations for resource-limited settings. *The Lancet Infectious diseas* **4**(3): 155-65.
16. Perez-Velez CM, Marais BJ. Tuberculosis in children. *The New England* *medicine* 2012; **367**(4): 348-61.
17. Pearce EC, Woodward JF, Nyandiko WM, Vreeman RC, Ayaya SO. A sy review of clinical diagnostic systems used in the diagnosis of tuberculosis in chil *research and treatment* 2012; **2012**: 401896.
18. Newton SM, Brent AJ, Anderson S, Whittaker E, Kampmann B. Paediatr tuberculosis. *The Lancet Infectious diseases* 2008; **8**(8): 498-510.
19. Mukherjee A, Lodha R, Kabra SK. Changing trends in childhood tubercu *journal of pediatrics* 2011; **78**(3): 328-33.
20. Marais BJ, Schaaf HS. Childhood tuberculosis: An emerging and previou neglected problem. *Infectious Disease Clinics of North America* 2010; **24**(3): 72[illegible]

21. Marais BJ, Rabie H, Cotton MF. TB and HIV in children - advances in prevention and management. *Paediatric respiratory reviews* 2011; **12**(1): 39-45.
22. Marais BJ, Graham SM, Cotton MF, Beyers N. Diagnostic and management challenges for childhood tuberculosis in the era of HIV. *The Journal of infectious diseases* 2007; **196 Suppl 1**: S76-85.
23. Maher D. Tuberculosis is important problem in children with HIV infection in sub-Saharan Africa. *BMJ (Clinical research ed)* 1996; **313**(7056): 562-3.
24. Link-Gelles R, Moultrie H, Sawry S, Murdoch D, Van Rie A. Tuberculosis Immune Reconstitution Inflammatory Syndrome in Children Initiating Antiretroviral Therapy for HIV Infection: A Systematic Literature Review. *The Pediatric infectious disease journal* 2014; **33**(5): 499-503.
25. Lalvani A, Millington KA. T cell-based diagnosis of childhood tuberculosis infection. *Current opinion in infectious diseases* 2007; **20**(3): 264-71.
26. Kaul D. Opportunistic infections in HIV infected children. *Indian Journal of Pediatrics* 2011; **78**(4): 471-2.
27. Jeena PM. The interaction of HIV and tuberculosis in childhood. *South African medical journal = Suid-Afrikaanse tydskrif vir geneeskunde* 2007; **97**(10 Pt 2): 989-91.
28. Hussain T. HIV infection among different population groups in India: A comprehensive review of seroprevalence and co-infection studies. *HIV and AIDS Review* 2014; **13**(3): 68-77.
29. Hoskyns W. Paediatric tuberculosis. *Postgraduate medical journal* 2003; **79**(931): 272-8.
30. Graham SM, Gibb DM. HIV disease and respiratory infection in children. *British medical bulletin* 2002; **61**: 133-50.
31. Graham SM. HIV and respiratory infections in children. *Current opinion in pulmonary medicine* 2003; **9**(3): 215-20.
32. Graham SM. Impact of HIV on childhood respiratory illness: differences between developing and developed countries. *Pediatric pulmonology* 2003; **36**(6): 462-8.
33. Fox GJ, Barry SE, Britton WJ, Marks GB. Contact investigation for tuberculosis: a systematic review and meta-analysis. *The European respiratory journal* 2013; **41**(1): 140-56.
34. Feja K, Saiman L. Tuberculosis in children. *Clinics in chest medicine* 2005; **26**(2): 295-vii.
35. du Cros P, Nyang'wa B-T, Gale M, Venis S, Ford N. Counting children: comparing reporting for paediatric HIV and tuberculosis. *Bulletin of the World Health Organization* 2011; **89**(12): 855.
36. Donald PR. Childhood tuberculosis: out of control? *Current opinion in pulmonary medicine* 2002; **8**(3): 178-82.
37. Donald PR. Childhood tuberculosis. *Current opinion in pulmonary medicine* 2000; **6**(3): 187-92.
38. Detjen AK, Magdorf K. Characteristics of childhood tuberculosis. *Pneumologie* 2009; **63**(4): 207-18.
39. Delacourt C. [Tuberculosis and HIV infection in children: a poorly controlled, high-risk situation]. *Tuberculose et infection VIH de l'enfant : une situation a haut risque, encore trop frequente* 2012; **29**(9): 1075-6.
40. Davidson RN. Childhood tuberculosis--problems ahead. *Transactions of the Royal Society of Tropical Medicine and Hygiene* 2000; **94**(1): 5-6.
41. Datta M, Swaminathan S. Global aspects of tuberculosis in children. *Paediatric respiratory reviews* 2001; **2**(2): 91-6.

42. Cotton MF, Rabie H, van Zyl GU. Antiretroviral therapy in children with tuberculosis: progress toward defining the issues. *The Journal of infectious diseases* 2010; **201**(8): 1113-4.
43. Correa AG. Unique aspects of tuberculosis in the pediatric population. *Clinics in chest medicine* 1997; **18**(1): 89-98.
44. Chisti MJ, Ahmed T, Pietroni MAC, et al. Pulmonary tuberculosis in severely-malnourished or HIV-infected children with pneumonia: a review. *Journal of health, population, and nutrition* 2013; **31**(3): 308-13.
45. Chintu C. Tuberculosis and human immunodeficiency virus co-infection in children: management challenges. *Paediatric respiratory reviews* 2007; **8**(2): 142-7.
46. Chamie G, Luetkemeyer A, Charlebois E, Havlir DV. Tuberculosis as part of the natural history of HIV infection in developing countries. *Clinical infectious diseases : an official publication of the Infectious Diseases Society of America* 2010; **50 Suppl 3**: S245-54.
47. Chakraborty R. Infections and other causes of death in HIV-infected children in Africa. *Paediatric Respiratory Reviews* 2004; **5**(2): 132-9.
48. Boulle A, Eley B. Commentary: Reducing HIV-associated tuberculosis in children. *International journal of epidemiology* 2009; **38**(6): 1621-3.
49. Berhan Y. Age and CD4 count of vertically HIV-infected children at the time of diagnosis: what are independent predictors for being symptomatic and CD4 counts drop? *Journal of tropical pediatrics* 2011; **57**(1): 14-23.
50. Bates M, Mudenda V, Mwaba P, Zumla A. Deaths due to respiratory tract infections in Africa: a review of autopsy studies. *Current opinion in pulmonary medicine* 2013; **19**(3): 229-37.
51. Ayieko J, Abuogi L, Simchowitz B, Bukusi EA, Smith AH, Reingold A. Efficacy of isoniazid prophylactic therapy in prevention of tuberculosis in children: a meta-analysis. *BMC infectious diseases* 2014; **14**: 91.
52. Adhikari M, Jeena P, Bobat R, et al. HIV-Associated Tuberculosis in the Newborn and Young Infant. *International journal of pediatrics* 2011; **2011**: 354208.
53. López-Varela E, Augusto O, Gondo K, et al. Incidence of tuberculosis in children under the age of three in Manhiça, rural southern Mozambique.
54. Kwara A, Enimil A, Gillani FS, et al. Pharmacokinetics of First-Line Antituberculosis Drugs Using WHO Revised Dosage in Children With Tuberculosis With and Without HIV Coinfection. *Journal of the Pediatric Infectious Diseases Society* 2015: piv035.
55. Perfura Yone EW, Mbarga AE, Kuaban C. Impact de l'infection à VIH sur la tuberculose de l'enfant à Yaoundé, Cameroun. *Revue des Maladies Respiratoires* 2012; **29**(9): 1095-103.
56. Jeena PM, Mitha T, Bamber S, Wesley A, Coutsoudis A, Coovadia HM. Effects of the human immunodeficiency virus on tuberculosis in children. *Tubercle and lung disease : the official journal of the International Union against Tuberculosis and Lung Disease* 1996; **77**(5): 437-43.
57. Crook AM, Turkova A, Musiime V, et al. Tuberculosis incidence is high in HIV-infected African children but is reduced by co-trimoxazole and time on antiretroviral therapy. *BMC Med* 2016; **14**: 50.
58. Viechtbauer W, others. Conducting meta-analyses in R with the metafor package. *Journal of Statistical Software* 2010; **36**(3): 1-48.
59. Team RC. R: A language and environment for statistical computing. R Foundation for Statistical Computing, Vienna, Austria. URL http://www.R-project.org.
60. Crook AM, Turkova A, Muslime V, et al. TB incidence is high in HIV--infected African children but is reduced by co--trimoxazole and time on antiretroviral therapy. *submitted* 2016.

61. Bates D, Maechler M, Bolker B, Walker S. lme4: Linear mixed-effects models using Eigen and S4. R package version 1.1-7. *This is computer program (R package) The URL of the package is:* http://CRAN *R-project org/package= lme4* 2014.
62. Abuogi LL, Mwachari C, Leslie HH, et al. Impact of expanded antiretroviral use on incidence and prevalence of tuberculosis in children with HIV in Kenya. *The international journal of tuberculosis and lung disease : the official journal of the International Union against Tuberculosis and Lung Disease* 2013; **17**(10): 1291-7.
63. Ade S, Harries AD, Trebucq A, et al. The burden and outcomes of childhood tuberculosis in Cotonou, Benin. *Public Health Action* 2013; **3**(1): 15-9.
64. Berggren Palme I, Gudetta B, Bruchfeld J, Eriksson M, Giesecke J. Detection of Mycobacterium tuberculosis in gastric aspirate and sputum collected from Ethiopian HIV-positive and HIV-negative children in a mixed in- and outpatient setting. *Acta paediatrica (Oslo, Norway : 1992)* 2004; **93**(3): 311-5.
65. Berggren Palme I, Gudetta B, Degefu H, Muhe L, Bruchfeld J, Giesecke J. A controlled estimate of the risk of HIV infection in Ethiopian children with tuberculosis. *Epidemiology and infection* 2001; **127**(3): 517-25.
66. Bhat GJ, Diwan VK, Chintu C, Kabika M, Masona J. HIV, BCG and TB in children: a case control study in Lusaka, Zambia. *Journal of tropical pediatrics* 1993; **39**(4): 219-23.
67. Bobossi-Serengbe G, Tembeti PJ, Mobima T, Yango F, Kassa-Kelembho E. [Tuberculosis and HIV co-infection among children hospitalized in Bangui (Central African Republic)]. *Co-infection tuberculose et virus de l'immunodeficience humaine chez les enfants hospitalises a Bangui (Republique Centrafricaine)* 2005; **12**(8): 1215-20.
68. Cathebras P, Vohito JA, Yete ML, et al. [Tuberculosis and human immunodeficiency virus infection in the Central African Republic]. *Tuberculose et infection par le virus de l'immunodeficience humaine en Republique Centrafricaine* 1988; **48**(4): 401-7.
69. Chintu C, Bhat G, Luo C, et al. Seroprevalence of human immunodeficiency virus type 1 infection in Zambian children with tuberculosis. *The Pediatric infectious disease journal* 1993; **12**(6): 499-504.
70. Chintu C, Dupont HL, Kaile T, et al. Human immunodeficiency virus-associated diarrhea and wasting in Zambia: selected risk factors and clinical associations. *The American journal of tropical medicine and hygiene* 1998; **59**(1): 38-41.
71. Chintu C, Luo C, Bhat G, et al. Impact of the human immunodeficiency virus type-1 on common pediatric illnesses in Zambia. *Journal of tropical pediatrics* 1995; **41**(6): 348-53.
72. Daniel OJ, Ogunfowora OB, Oladapo OT. HIV sero-prevalence among children diagnosed with TB in Nigeria. *Tropical doctor* 2007; **37**(4): 268-9.
73. Edwards DJ, Kitetele F, Van Rie A. Agreement between clinical scoring systems used for the diagnosis of pediatric tuberculosis in the HIV era. *The international journal of tuberculosis and lung disease : the official journal of the International Union against Tuberculosis and Lung Disease* 2007; **11**(3): 263-9.
74. Espinal MA, Reingold AL, Perez G, et al. Human immunodeficiency virus infection in children with tuberculosis in Santo Domingo, Dominican Republic: prevalence, clinical findings, and response to antituberculosis treatment. *Journal of acquired immune deficiency syndromes and human retrovirology : official publication of the International Retrovirology Association* 1996; **13**(2): 155-9.
75. Feldacker C, Tweya H, Keiser O, et al. Characteristics of adults and children diagnosed with tuberculosis in Lilongwe, Malawi: findings from an integrated HIV/TB clinic. *Tropical medicine & international health : TM & IH* 2012; **17**(9): 1108-16.
76. Gava C, Malacarne J, Rios DPG, Sant'Anna CC, Camacho LAB, Basta PC. Tuberculosis in indigenous children in the Brazilian Amazon. *Revista de saude publica* 2013; **47**(1): 77-85.

77. Geoghagen M, Farr JA, Hambleton I, Pierre R, Christie CDC. Tuberculosis and HIV co-infections in Jamaican children. *The West Indian medical journal* 2004; **53**(5): 339-45.
78. Henegar C, Behets F, Vanden Driessche K, Tabala M, Van Rie A. Impact of HIV on clinical presentation and outcomes of tuberculosis treatment at primary care level. *The international journal of tuberculosis and lung disease : the official journal of the International Union against Tuberculosis and Lung Disease* 2013; **17**(11): 1411-3.
79. Hesseling AC, Cotton MF, Jennings T, et al. High incidence of tuberculosis among HIV-infected infants: evidence from a South African population-based study highlights the need for improved tuberculosis control strategies. *Clinical infectious diseases : an official publication of the Infectious Diseases Society of America* 2009; **48**(1): 108-14.
80. Iriso R, Mudido PM, Karamagi C, Whalen C. The diagnosis of childhood tuberculosis in an HIV-endemic setting and the use of induced sputum. *The international journal of tuberculosis and lung disease : the official journal of the International Union against Tuberculosis and Lung Disease* 2005; **9**(7): 716-26.
81. Llerena C, Fadul SE, Garzon MC, et al. [Drug-resistant Mycobacterium tuberculosis in children under 15 years]. *Resistencia de Mycobacterium tuberculosis a los farmacos antituberculosos en menores de 15 anos en Colombia* 2010; **30**(3): 362-70.
82. Luo C, Chintu C, Bhat G, et al. Human immunodeficiency virus type-1 infection in Zambian children with tuberculosis: changing seroprevalence and evaluation of a thioacetazone-free regimen. *Tubercle and lung disease : the official journal of the International Union against Tuberculosis and Lung Disease* 1994; **75**(2): 110-5.
83. Madhi SA, Gray GE, Huebner RE, Sherman G, McKinnon D, Pettifor JM. Correlation between CD4+ lymphocyte counts, concurrent antigen skin test and tuberculin skin test reactivity in human immunodeficiency virus type 1-infected and -uninfected children with tuberculosis. *The Pediatric infectious disease journal* 1999; **18**(9): 800-5.
84. Mehta S, Mugusi FM, Bosch RJ, et al. A randomized trial of multivitamin supplementation in children with tuberculosis in Tanzania. *Nutrition journal* 2011; **10**: 120.
85. Miranda AE, Dietze R, Maciel EL, et al. Tuberculosis and AIDS co-morbidity in children: linkage of databases from Espirito Santo State, Brazil. *Journal of tropical pediatrics* 2011; **57**(4): 296-8.
86. Mukadi YD, Wiktor SZ, Coulibaly IM, et al. Impact of HIV infection on the development, clinical presentation, and outcome of tuberculosis among children in Abidjan, Cote d'Ivoire. *AIDS (London, England)* 1997; **11**(9): 1151-8.
87. Berggren Palme I, Gudetta B, Bruchfeld J, Muhe L, Giesecke J. Impact of human immunodeficiency virus 1 infection on clinical presentation, treatment outcome and survival in a cohort of Ethiopian children with tuberculosis. *The Pediatric infectious disease journal* 2002; **21**(11): 1053-61.
88. Rachow A, Clowes P, Saathoff E, et al. Increased and expedited case detection by Xpert MTB/RIF assay in childhood tuberculosis: a prospective cohort study. *Clinical infectious diseases : an official publication of the Infectious Diseases Society of America* 2012; **54**(10): 1388-96.
89. Ramos JM, Reyes F, Tesfamariam A. Childhood and adult tuberculosis in a rural hospital in Southeast Ethiopia: a ten-year retrospective study. *BMC public health* 2010; **10**: 215.
90. Rose MV, Kimaro G, Nissen TN, et al. QuantiFERON-TB gold in-tube performance for diagnosing active tuberculosis in children and adults in a high burden setting. *PloS one* 2012; **7**(7): e37851.
91. Sassan-Morokro M, De Cock KM, Ackah A, et al. Tuberculosis and HIV infection in children in Abidjan, Cote d'Ivoire. *Transactions of the Royal Society of Tropical Medicine and Hygiene* 1994; **88**(2): 178-81.

92. Schaaf HS, Hesseling AC, Rautenbach C, Seddon JA. Trends in childhood drug-resistant tuberculosis in South Africa: a window into the wider epidemic? *The international journal of tuberculosis and lung disease : the official journal of the International Union against Tuberculosis and Lung Disease* 2014; **18**(7): 770-3.
93. Seddon JA, Hesseling AC, Marais BJ, Jordaan A, Victor T, Schaaf HS. The evolving epidemic of drug-resistant tuberculosis among children in Cape Town, South Africa. *The international journal of tuberculosis and lung disease : the official journal of the International Union against Tuberculosis and Lung Disease* 2012; **16**(7): 928-33.
94. Thomas TA, Heysell SK, Moodley P, et al. Intensified specimen collection to improve tuberculosis diagnosis in children from Rural South Africa, an observational study. *BMC infectious diseases* 2014; **14**: 11.
95. Yassin MA, Petrucci R, Garie KT, et al. Can interferon-gamma or interferon-gamma-induced-protein-10 differentiate tuberculosis infection and disease in children of high endemic areas? *PloS one* 2011; **6**(9): e23733.
96. Bakeera-Kitaka S, Conesa-Botella A, Dhabangi A, et al. Tuberculosis in human immunodeficiency virus infected Ugandan children starting on antiretroviral therapy. *The international journal of tuberculosis and lung disease : the official journal of the International Union against Tuberculosis and Lung Disease* 2011; **15**(8): 1082-6.
97. Braitstein P, Nyandiko W, Vreeman R, et al. The clinical burden of tuberculosis among human immunodeficiency virus-infected children in Western Kenya and the impact of combination antiretroviral treatment. *The Pediatric infectious disease journal* 2009; **28**(7): 626-32.
98. Edmonds A, Lusiama J, Napravnik S, Kitetele F, Van Rie A, Behets F. Anti-retroviral therapy reduces incident tuberculosis in HIV-infected children. *International journal of epidemiology* 2009; **38**(6): 1612-21.
99. Li N, Manji KP, Spiegelman D, et al. Incident tuberculosis and risk factors among HIV-infected children in Tanzania. *AIDS (London, England)* 2013; **27**(8): 1273-81.
100. Martinson NA, Moultrie H, van Niekerk R, et al. HAART and risk of tuberculosis in HIV-infected South African children: a multi-site retrospective cohort. *The international journal of tuberculosis and lung disease : the official journal of the International Union against Tuberculosis and Lung Disease* 2009; **13**(7): 862-7.
101. Yirdaw KD, Jerene D, Gashu Z, et al. Beneficial effect of isoniazid preventive therapy and antiretroviral therapy on the incidence of tuberculosis in people living with HIV in Ethiopia. *PloS one* 2014; **9**(8): e104557.

First author/year	incidence <6wks	incidence 6wks-6mo	incidence <2/12	incidence 2-6/12	incidence within 6/12	incidence >6/12	incidence within 3/12 art	incidence 3-6/12 art	incidence 6-12/12	incidence 12-18/12	incidence 18-24/12	incidence 13-24/12	incidence 25-36/12	incidence >3/12	incidence >12/12 art
Abuogi, 2013															
Alarcon, 2012															
Auld, 2014						0.56(0.36-0.89)	6.28(4.31-9.16)	2.52(1.36-4.69)							
Bakeera-Kitaka, 2011	34.5(25.9-46.1)	27.0(21.5-34.6)							10.8(8.3-14.4)						7.4(4.9-11.0)
Braitstein, 2009			355 (321.1-392.3)	46 (40.1-52.8)		4.7(4.0-5.4)									
Brennan, 2014					5.9 (4.7-7.4)	2.1 (1.4-3.0)									
Ciaranello, 2014															
Crook, 2016															
Curtis, 2012							27.3(26.3-28.4)	11.9(11.1-12.8)	6.1(5.7-6.5)			3.6 (3.3-3.8)	3.7(3.4-4.1)		
Dankne, 2001															
De Beaudrap, 2013					8.1(3.9-17)				3.8(2.0-10.4)	3.3(2.6-10.2)	4.3(3.2-11.9)				
Edmonds, 2009					18.9(11.6-29.2)				11.4(5.5-21.0)						5.3(2.7-9.5)
Gray, 2014															
Kouakoussui, 2004															
Li, 2013						2.3 (1.9-2.7)	15.0 (12.7-17.7)	7.0(5.8-8.5)							
Martinson, 2009															
Prasitsuebsai, 2014															
Thomas, 2000															
Walters, 2008															
Walters, 2014							21.9(13.9-32.9)							3.9(2.7-5.5)	
Yirdaw, 2014															
Zar, 2007															

First author/year	relative incidence ns	relative incidence mild	relative incidence advanced	relative incidence sev	CD4%<5	5<CD4%<15	15<CD4%	CD4% <10	10<=CD4%<20	20<=CD4%	CD4% <15	15<=CD4%<24	25<CD4%	25<=CD4%	CD4>250	250>CD4>200	CD4<200	incidence <12 wks	incidence >12 <52 wks	incidence >52 wks
Abuogi, 2013	1	6.09 (0.88-42.25)	17.2 (3.18-25.71)	9.16 (1.28-65.74)																
Alarcon, 2012																				
Auld, 2014								1.86 (0.42-8.23)	1.75 (0.41-7.40)	1										
Bakeera-Kitaka, 2011																				
Braitstein, 2009				HR: 4.44 (3.62-5.44) vs. not severely immunosuppressed																
Brennan, 2014													4.8 (3.2-7.0)	5.7 (3.9-8.2)						
Ciaranello, 2014											37.3 (28.2-48.5)	17 (12.7-22.2)	8.7 (5.9-12.5)							
Crook, 2016																		8.8 (5.2-13.4)	2.7 (1.7-4.3)	1.2 (0.8-1.7)
Curtis, 2012																				
Dankne, 2001											0.6 (0.3-1.0)	0.3 (0.1-0.7)	0.2 (0.1-0.4)							
De Beaudrap, 2013											6.3(2.7-12.5)	1.32(0.2-4.8)	0(0-4.7)							
Edmonds, 2009	1	1.21 (0.53-2.75)	1.52 (0.61-3.80)	7.53 (3.92-14.49)																
Gray, 2014																				
Kouakoussui, 2004					9.7 (3.7-21.3)	2.6 (0.5-8.2)	0 (0-8.8)													
Li, 2013	1	included in left	1.6 (1.2-2.4)	2.6 (1.8-3.8)																
Martinson, 2009															1.17(0.19-2.15)	0.89(0.15-1.62)	1			
Prasitsuebsai, 2014																				
Thomas, 2000																				
Walters, 2008																				
Walters, 2014																				
Yirdaw, 2014																				
Zar, 2007																				

First author/year	incidence <6wks	incidence 6wks-6mo	incidence <2/12	incidence 2-6/12	incidence within 6/12	incidence >6/12	incidence within 3/12 art	incidence 3-6/12 art	incidence 6-12/12	incidence 12-18/12	incidence 18-24/12	incidence 13-24/12	incidence 25-36/12	incidence >3/12	incidence >12/12 art	relative incidence ns	relative incidence mild	relative incidence advanced	relative incidence sev	CD4%<5	5<CD4%<15	15<CD4%	CD4% <10	10<=CD4%<20	20<=CD4%	CD4% <15	15<=CD4%<24	25<CD4%	25<=CD4%	CD4>250	250>CD4>200	CD4<200	incidence <12 wks	incidence >12 <52 wks	incidence >52 wks
Abuogi, 2013																1	6.09 (0.88- 42.25)	17.2 (3.18- 25.71)	9.16 (1.28-65.74)																
Alarcon, 2012																																			
Auld, 2014						0.56(0.36 -0.89)	6.28(4.31 -9.16)	2.52(1.36 -4.69)															1.86 (0.42 - 8.23)	1.75 (0.41 - 7.40)	1										
Bakeera-Kitaka, 2011	34.5(25.9 -46.1)	27.0(21.5 -34.6)							10.8(8.3 -14.4)						7.4(4.9 -11.0)																				
Braitstein, 2009			355 (321.1 - 392.3)	46 (40.1 - 52.8)		4.7(4.0- 5.4)													HR: 4.44 (3.62- 5.44) vs. not severely immunosuppressed																
Brennan, 2014					5.9 (4.7- 7.4)	2.1 (1.4- 3.0)																						4.8 (3.2- 7.0)	5.7 (3.9 - 8.2)						
Ciaranello, 2014																										37.3 (28.2- 48.5)	17 (12.7- 22.2)	8.7 (5.9- 12.5)							
Crook, 2016																																	8.8 (5.2- 13.4)	2.7 (1.7 - 4.3)	1.2 (0.8 - 1.7)
Curtis, 2012							27.3(26.3 -28.4)	11.9(11.1 -12.8)	6.1(5.7- 6.5)			3.6 (3.3 - 3.8)	3.7(3.4 -4.1)																						
Dankne, 2001																										0.6 (0.3- 1.0)	0.3 (0.1- 0.7)	0.2 (0.1- 0.4)							
De Beaudrap, 2013					8.1(3.9- 17)				3.8(2.0- 10.4)	3.3(2.6 -10.2)	4.3(3.2 -11.9)															6.3(2.7 -12.5)	1.32(0.2 -4.8)	0(0- 4.7)							
Edmonds, 2009					18.9(11.6 -29.2)				11.4(5.5 -21.0)						5.3(2.7 -9.5)	1	1.21 (0.53- 2.75)	1.52 (0.61- 3.80)	7.53 (3.92-14.49)																
Gray, 2014																																			
Kouakoussui, 2004																				9.7 (3.7- 21.3)	2.6 (0.5 - 8.2)	0 (0- 8.8)													
Li, 2013						2.3 (1.9- 2.7)	15.0 (12.7- 17.7)	7.0(5.8- 8.5)								1	included in left	1.6 (1.2- 2.4)	2.6 (1.8-3.8)																
Martinson, 2009																														1.17(0.19 -2.15)	0.89(0.15 -1.62)	1			
Prasitsuebsai, 2014																																			
Thomas, 2000																																			
Walters, 2008																																			
Walters, 2014							21.9(13.9 -32.9)							3.9(2.7 -5.5)																					
Yirdaw, 2014																																			
Zar, 2007																																			
